# Bone-organ axes: bidirectional crosstalk

**DOI:** 10.1186/s40779-024-00540-9

**Published:** 2024-06-12

**Authors:** An-Fu Deng, Fu-Xiao Wang, Si-Cheng Wang, Ying-Ze Zhang, Long Bai, Jia-Can Su

**Affiliations:** 1 Institute of Translational Medicine, Organoid Research Center, Shanghai University, Shanghai, 200444 China; 2 National Center for Translational Medicine (Shanghai) SHU Branch, Shanghai University, Shanghai, 200444 China; 3 Department of Orthopedics, Shanghai Zhongye Hospital, Shanghai, 200444 China; 4 Department of Orthopaedics, the Third Hospital of Hebei Medical University, Orthopaedic Research Institution of Hebei Province, NHC Key Laboratory of Intelligent Orthopaedic Equipment, Shijiazhuang, 050051 China; 5 School of Medicine, Shanghai University, Shanghai, 200444 China; 6 Wenzhou Institute of Shanghai University, Wenzhou, 325000 Zhejiang China; 7 Department of Orthopaedics, Xinhua Hospital Affiliated to Shanghai Jiao Tong University School of Medicine, Shanghai, 200092 China

**Keywords:** Bone-organ axes, Bidirectional crosstalk, Cytokines, Osteokines, Extracellular vesicles, Hormones, Metabolites

## Abstract

In addition to its recognized role in providing structural support, bone plays a crucial role in maintaining the functionality and balance of various organs by secreting specific cytokines (also known as osteokines). This reciprocal influence extends to these organs modulating bone homeostasis and development, although this aspect has yet to be systematically reviewed. This review aims to elucidate this bidirectional crosstalk, with a particular focus on the role of osteokines. Additionally, it presents a unique compilation of evidence highlighting the critical function of extracellular vesicles (EVs) within bone-organ axes for the first time. Moreover, it explores the implications of this crosstalk for designing and implementing bone-on-chips and assembloids, underscoring the importance of comprehending these interactions for advancing physiologically relevant in vitro models. Consequently, this review establishes a robust theoretical foundation for preventing, diagnosing, and treating diseases related to the bone-organ axis from the perspective of cytokines, EVs, hormones, and metabolites.

## Background

The intricate network of crosstalk between organs is a fundamental aspect of physiological homeostasis and disease adaptation. Within the human body, bone serves not only as a structural support but also as an active participant in endocrine regulation through the secretion of bone-derived cytokines, known as “osteokines” [1]. Although the influence of bone on other organs has been extensively studied and reviewed [2], there has been a significant oversight regarding the reciprocal influence of these organs on bone physiology. As a result, there is currently a lack of comprehensive summarization and systematic analysis in this area. This bidirectional communication is referred to as the bone-organ axis. For example, in the brain-bone axis, while the brain exerts dominant effects on bone metabolism, homeostasis, and disease progression; conversely, bones also signal to the brain to promote brain development and skeletal growth. Understanding this bidirectional communication is crucial for comprehending the pathophysiology of bone diseases and systemic disorders.

The bidirectional crosstalk between bone and other organs primarily occurs through the secretion of osteokines, hormones, and metabolites. For instance, osteocalcin (OCN), an osteokine synthesized by osteoblasts, plays a pivotal role in the maturation and functioning of reproductive organs [3, 4]. Conversely, reproductive hormones such as testosterone and estrogen influence the bone remodeling and integrity [5].

Extracellular vesicles (EVs), a distinct category of lipid bilayer-bound particles naturally released by almost all cell types, have recently garnered considerable attention as potent mediators in intercellular communication [6]. In the growing field of research on their multifaceted capabilities, EVs have been implicated in diverse processes, ranging from immune modulation to tissue regeneration and even tumor progression [7–9]. Notably, recent finding has shed light on the profound impact of EVs on bone-organ crosstalk [10]. EVs secreted by bone-related cells have been shown to regulate the metabolic activities of distant organs, including the liver [11, 12], adipose tissue [13], and muscle [14]. Reciprocally, brain-derived EVs containing microRNA (miR)-483-5p [15] and adipose tissue-derived EVs carrying miR-181a and miR-181b [16] influence bone homeostasis, highlighting their crucial significance in bone-organ crosstalk. Fundamentally, osteokines and EVs significantly orchestrate the bidirectional crosstalk within bone-organ axes, paving the way for novel insights into systemic physiology and disease.

**Fig. 1:**
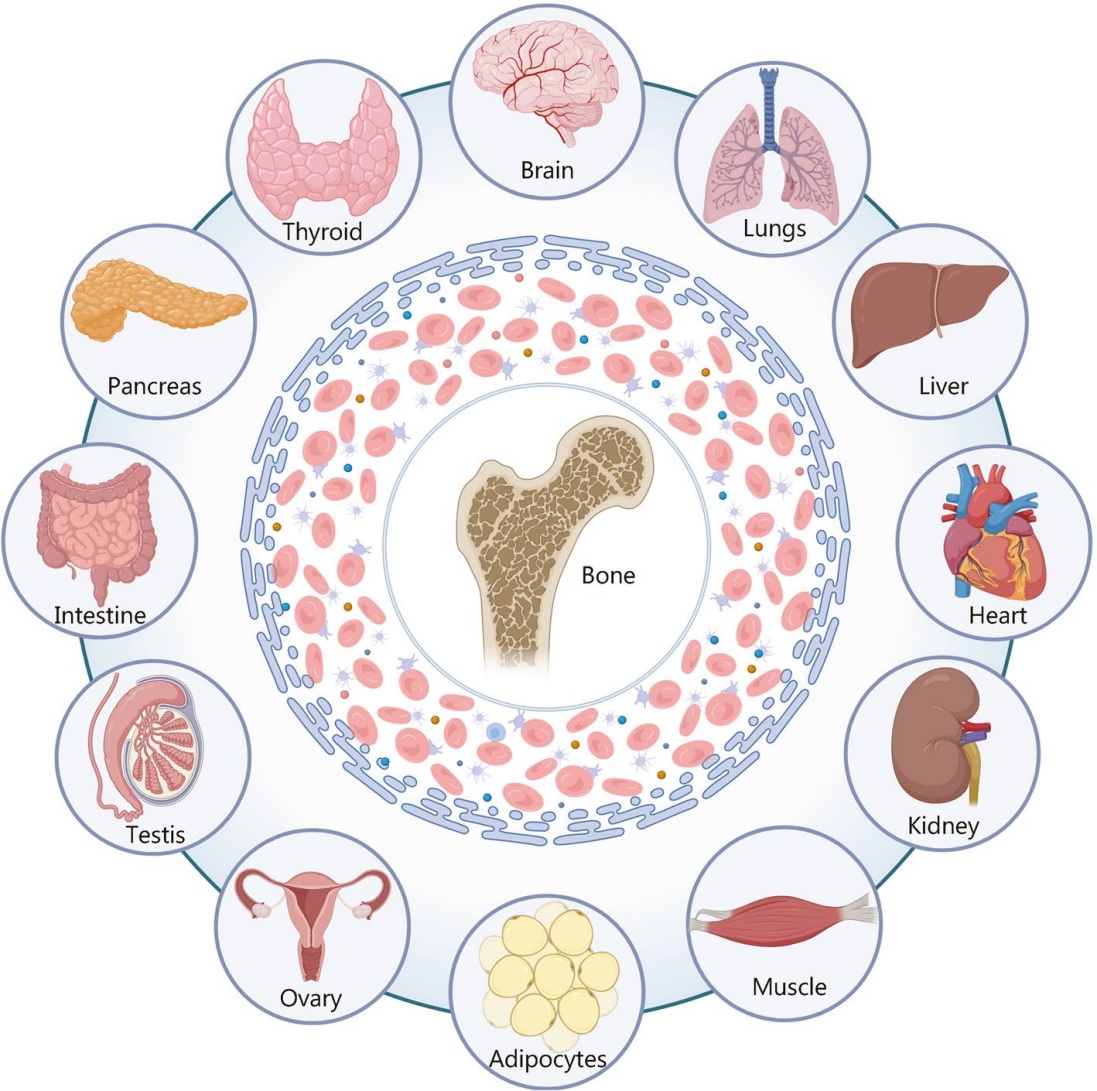
Bone-organ axes: bidirectional crosstalk

This review aims to comprehensively elucidate the bidirectional crosstalk between bone and other organs (Fig. 1), thereby providing insights into the treatment of bone-related diseases and systemic disorders. Furthermore, it briefly discusses the significance of unraveling bone-organ axes in the context of organ-on-chip, organoid, and assembloids research. Understanding these intricate interactions is crucial for developing not only more sophisticated and physiologically relevant in vitro models but also potential therapeutic applications for addressing bone-related and systemic diseases.

## Bone-organ axes

### Brain-bone axis: neuroendocrine regulation and skeletal health

The concept of the brain-bone axis refers to a bidirectional communication network between the central nervous system (CNS) and skeletal system, elucidating the intricate physiological interplay that underpins bone homeostasis and neuroregulation. Emerging evidence suggests that this axis encompasses not only hormonal and neuronal pathways but also integrates complex molecular and cellular mechanisms, highlighting the pivotal role of the CNS in skeletal health and disease [17–19]. Significant advancements in neuroscience and bone biology have shed light on the molecular foundations of this axis, revealing how central signals can influence processes involved in bone remodeling, such as osteoblastogenesis and osteoclastogenesis, thereby impacting bone density, structure, and overall health. The hypothalamus directly affects osteoblast activity and bone formation through the secretion of hormones like leptin, emphasizing a crucial connection between metabolic states and bone density. Moreover, the discovery of OCN as a hormone derived from bones has further demonstrated the bone’s role as an endocrine organ capable of influencing brain function and systemic energy metabolism [20]. Exploring the brain-bone axis opens up new avenues for understanding the pathophysiology of conditions like osteoporosis, arthritis, and other metabolic bone diseases while presenting potential therapeutic targets that leverage the neuroskeletal interface.

#### Brain to bone: neurological influence on skeletal integrity

**Fig. 2:**
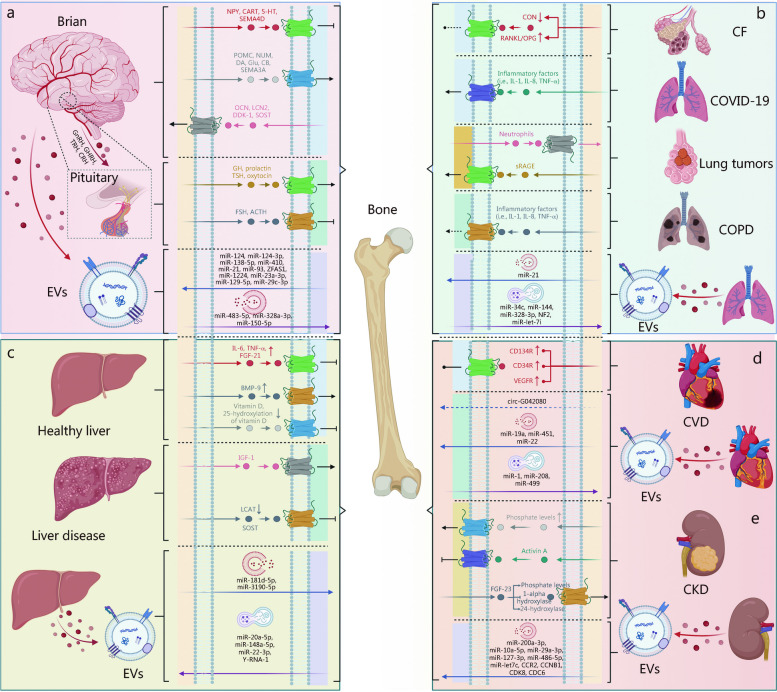
Interconnected networks of the bone-organ axes. This figure illustrates the complex network of communication pathways between the skeletal system and major organs, encompassing both physiological and pathological states. **a** Within the bone-brain axis, a diverse array of molecules such as neuropeptides, neurotransmitters, and EVs orchestrate the communication between the brain and bone, underpinning cognitive functions and skeletal health. **b** The lung-bone axis unravels how pulmonary processes and bone remodeling influence each other via osteokines, inflammatory markers, and EVs, with implications for conditions ranging from cystic fibrosis (CF) to chronic obstructive pulmonary disease (COPD). **c** The liver-bone axis sheds light on the reciprocal interactions through hepatokines, osteokines, and EVs, which are pivotal in maintaining metabolic balance and responding to liver disease. **d** The heart-bone axis reveals the bidirectional communication, where cardiac markers and EVs reflect the interplay affecting cardiovascular health. **e** The kidney-bone axis focuses on the regulatory role of the kidneys in mineral and bone homeostasis, particularly through the modulation of phosphate and calcium levels, and the systemic implications of chronic kidney disease (CKD) on bone architecture. Each axis portrays a specific organ-bone interaction spectrum, from molecular signals to disease, providing a comprehensive view of the bone’s central role in systemic physiology and pathology. GnRH gonadotropin-releasing hormone, GHRH growth hormone-releasing hormone, TRH thyrotropin-releasing hormone, CRH corticotropin-releasing factor, NPY neuropeptide Y, CART cocaine amphetamine-regulated transcript, 5-HT 5-hydroxytryptamine, SEMA4D semaphoring 4D, SEMA3A semaphoring 3A, POMC proopiomelanocortin, NMU neuromedin U, DA dopamine, Glu glutamate, CB cannabinoid, OCN osteocalcin, LCN2 lipocalin 2, DKK-1 Dickkopf 1, SOST sclerostin, GH growth hormone, TSH thyroid stimulating hormone, FSH follicle-stimulating hormone, ACTH adrenocorticotropic hormone, IL-1/6/8 interleukin-1/6/8, TNF-α tumor necrosis factor-α, BMP-9 bone morphogenetic protein-9, IGF-1 insulin-like growth factor 1, LCAT lecithin-cholesterol acyltransferase, RANKL receptor activator of nuclear factor kappa-B ligand, OPG osteoprotegerin, sRAGE soluble receptors for advanced glycation end products, PTH parathyroid hormone, FGF-21/23 fibroblast growth factor-21/23, COVID-19 chronic coronavirus disease 2019, EVs extracellular vesicles, CVD cardiovascular disease, CCR2 C–C motif chemokine receptor 2, CCNB1 cyclin B1, CDK8 cyclin-dependent kinase 8, CDC6 cell division cycle 6, NF2 neurofibromin 2, VEGFR vascular endothelial growth factor receptor

The brain plays a dominant role in regulating bone metabolism, maintaining homeostasis, and influencing disease progression. Conversely, bone communicates with the brain to facilitate brain development and promote bone growth. This section describes the impact of the brain on bone through various neuropeptides [such as neuropeptide Y (NPY), cocaine amphetamine-regulated transcript (CART), proopiomelanocortin (POMC), and neuromedin U (NMU)], neurotransmitters [including 5-hydroxytryptamine (5-HT, serotonin), dopamine (DA), and excitatory neurotransmitter glutamate (Glu)], cannabinoids (CBs), semaphorins (Semas), as well as EVs produced by the brain (Fig. 2a, Table 1 [21–71]).

**Table 1:** Neuroendocrine and osteokines in the brain-bone axis associated with bone metabolism and neuroregulation

Factor	Source	Target cells	Effect	Function and application	References
Brain to bone					
Neuropeptide Y (NPY)	Central and peripheral nervous systems	Osteoblasts and osteoclasts	Inhibits osteoblast activity, stimulates osteoclastogenesis	Regulates bone mass and energy balance, contributing to the anabolic processes in bone formation	[21, 22]
Cocaine amphetamine-regulated transcript (CART)	Brain	Osteoblasts	Promotes osteoblast differentiation and proliferation	Involved in bone formation and energy homeostasis	[23, 24]
5-hydroxytryptamine (5-HT, serotonin)	Brain, gut	Osteoblasts, osteoclasts	Modulates osteoblast and osteoclast activity	Influences bone mass and remodeling, with effects depending on its site of production. 5-HT produced by peripheral nerves inhibits bone formation, while 5-HT produced by central nerves promotes bone formation	[25–28]
Semaphorins (Semas)	Brain	Brain	Influence nerve growth, regulate osteoclast and osteoblast differentiation	Semas links bone metabolism with neurological functions. SEMA3A has a dual regulatory effect on osteoblasts and osteoclasts since it can promote osteoblast activity and inhibit osteoclast activity. SEMA4D inhibits osteoblast formation and mineralization and induces osteoclast formation during bone resorption	[29–31]
Proopiomelanocortin (POMC)	Brain	Osteoblasts, cartilage	Enhances osteoblast proliferation	Plays a role in bone growth and regeneration	[32–34]
Neuromedin U (NMU)	Brain, gut	Osteoblasts	Stimulates osteoblast proliferation and differentiation	Facilitates bone formation and enhanced bone matrix production and mineralization	[35, 36]
Dopamine (DA)	Brain	Osteoclasts	Inhibits osteoclast activity	Affect positively bone density and turnover	[37–40]
Glutamate (Glu)	Brain	Osteoblasts, osteoclasts	Inhibits osteoclast activity and promotes osteoblast function	Maintain bone homeostasis	[41–45]
Cannabinoids (CBs)	Brain	Osteoblasts, osteoclasts	Affects osteoblast and osteoclast activity, influences bone turnover	Modulates bone metabolism. Agonists and antagonists of CB1 and CB2 have great potential in clinical bone repair therapy	[46–49]
Growth hormone (GH)	Hypothalamus	Osteoblasts	Stimulates IGF-1 production; promotes osteoblast differentiation and proliferation	Enhances bone growth and density by stimulating bone formation; regulates longitudinal growth and bone mass	[50]
Follicle-stimulating hormone (FSH)	Hypothalamus	Ovaries, testes, osteoclasts	Increases estrogen and testosterone production; may enhance osteoclast differentiation	Indirectly influences bone density through regulation of sex steroids	[51–53]
Thyroid stimulating hormone (TSH)	Hypothalamus	Thyroid gland, osteoblasts, osteoclasts	Stimulates thyroid hormone (TH) production; modulates osteoblast and osteoclast activity	Regulates bone turnover and density through THs; hyperthyroidism can lead to increased bone resorption	[54–56]
Adrenocorticotropic hormone (ACTH)	Hypothalamus	Adrenal cortex, osteoblasts	Stimulates cortisol production; influences osteoblast differentiation	High levels of ACTH can lead to decreased bone formation	[57–59]
Bone to brain					
Osteocalcin (OCN)	Bone	Pancreatic β cells, brain, gonads	Increases insulin secretion, affects cognitive functions, influences testosterone production	Links bone metabolism with glucose homeostasis, cognitive function, and fertility	[60, 61]
Lipocalin 2 (LCN2)	Bone	Hypothalamus	Mediate the anorexia pathway to regulate energy metabolism binding to MC4R	Bone homeostasis is biased towards bone formation in MC4R-deficient mouse model	[62–64]
Sclerostin (SOST)	Bone	Osteoblasts	Inhibits osteoblast activity and bone formation	Regulates bone remodeling and density, antagonist of Wnt signaling in bone formation	[65–68]
Dickkopf-1 (DKK1)	Bone	Osteoblasts	Inhibits osteoblast activity by antagonizing Wnt signaling	Controls bone remodeling and development, involved in the pathogenesis of neurodegenerative diseases to bone	[69–71]

*IGF-1* insulin-like growth factor-1, *MC4R* melanocortin 4 receptor

NPY, a neuropeptide that regulates bone homeostasis, is expressed in the central and peripheral nervous systems to modulate the activity of osteoblasts and osteoclasts. Orexigenic NPY interacts with 5 distinct receptors [Y1 (NPY1R), Y2 (NPY2R), Y4 (NPY4R), Y5 (NPY5R), and Y6 (NPY6R)] to regulate multiple physiological functions. It exerts its influence on bone formation by suppressing osteoblast activity and promoting osteoclastogenesis through its Y1 and Y2 receptors. A study conducted in mice has reported that NPY binding to the osteoblast Y1 receptor enhances proliferation of mesenchymal stem cells (MSCs) and osteoprogenitor proliferation while inhibiting bone formation [21]. In a NPY2R-deficient mouse model, there was observed a bias towards increased bone formation, indicating an important role for this receptor in maintaining bone homeostasis [22].

Predominantly recognized for its influence on neuroendocrine functions related to appetite suppression and energy homeostasis, CART consists of two peptides (CART I and CART II) and extends its physiological impact on skeletal health by promoting osteoblast differentiation and proliferation [23]. The mechanisms through which CART exerts its osteogenic effects involve the modulation of crucial signaling pathways, including Wnt and bone morphogenetic proteins (BMPs), which are vital for osteoblast function and bone homeostasis. Given its dual role in energy regulation and bone metabolism, CART emerges as a potential therapeutic target for addressing bone diseases such as osteoporosis, offering a novel approach to enhancing bone density and strength through the manipulation of neuroendocrine pathways [23, 24].

5-HT is biosynthesized in neurons within the CNS and has also been detected in the skeletal system [25]. It can bind to receptors on osteoblasts and osteoclasts, thereby influencing bone homeostasis [26]. Different sources of 5-HT have varying effects on bone metabolism [27]. Peripheral nerve-derived 5-HT inhibits bone formation, while central nerve-derived 5-HT promotes it [27]. Clinical evidence supports these findings, demonstrating accelerated bone loss and reduced bone mass gain in postmenopausal women prescribed selective serotonin reuptake inhibitors [28]. Similarly, Semas are a family of cell surface and soluble proteins that include 3A (SEMA3A) and 4D (SEMA4D). They transduce cell signals and regulate cell differentiation and function [29]. Semas and their receptors are expressed both within the nervous system as well as outside it. SEMA3A exerts a dual regulatory effect on osteoblasts and osteoclasts by promoting osteoblast activity while inhibiting osteoclast activity [30]. Moreover, SEMA4D suppresses osteoblast formation and mineralization while inducing osteoclast formation during bone resorption [31].

In addition to the aforementioned factors, we briefly explore other key elements contributing to the complex brain-bone axis. POMC, a precursor protein, generates multiple peptides including adrenocorticotropic hormone (ACTH) and melanocyte-stimulating hormones, which possess immunomodulatory functions as well as protective effects on bone and cartilage [32, 33]. Specifically, the absence of estrogen receptors in POMC-expressing neurons has been shown to increase cortical bone mass in female mice, highlighting the negative impact of estrogen on bone through POMC-expressing neurons [34]. NMU is a neuropeptide with widespread expression, particularly in the gastrointestinal tract and CNS, implicating its multifunctionality across various physiological domains such as energy homeostasis regulation and stress responses [35]. NMU primarily stimulates osteoblast proliferation and differentiation to promote bone formation. This effect is mediated by binding to specific receptors (NMUR1 and NMUR2), expressed on osteoblasts, triggering intracellular signaling cascades that enhance bone matrix production and mineralization [36]. Neurotransmitters also play a crucial role. For example, DA inhibits osteoclast generation through its receptors DRD1 – DRD3 [37, 38] and DRD5 [39], positively influencing bone mass [40]. This is particularly relevant considering the decreased levels of DA observed in conditions such as Alzheimer’s disease, Parkinson’s disease, and depression, which could have secondary implications for bone health. Glu, another neurotransmitter, is involved in maintaining bone homeostasis by binding to its ionotropic receptors on both osteoblasts [41] and osteoclasts [42, 43]. Activation of these receptors inhibits osteoclast activity [44] while promoting osteoblast function [45], offering a balanced regulatory mechanism. CBs introduce an additional layer of intricacy, as they primarily bind to two receptor subtypes: CBR1 and CBR2. While CBR1 is mainly expressed in the CNS and stimulates bone resorption [46], CBR2 is primarily found in the peripheral nervous system and promotes bone formation by inhibiting receptor activator of nuclear factor kappa-B ligand (RANKL) [47]. In clinical studies, activation of CBR1 has been shown to inhibit the release of norepinephrine, a transmitter of the sympathetic nervous system, thereby stimulating bone formation [48]. Conversely, deficiencies in the gene encoding CBR2 have been associated with low bone mineral density (BMD) and osteoporosis [49]. Therefore, agonists and antagonists targeting CB1 and CB2 receptors hold significant potential for clinical applications in bone repair therapy.

The pituitary gland is situated below the hypothalamus. Its particular anatomical location results in direct regulation of most hormones secreted by the pituitary gland in the brain (Fig. 2a, Table 1). Among them, we examine the impact of growth hormone (GH), follicle-stimulating hormone (FSH), thyroid-stimulating hormone (TSH), and ACTH on bone metabolism.

Regulated by hypothalamic secretion of GH-releasing hormone (GHRH), GH is a bone regulatory factor produced by the anterior pituitary gland that exerts a positive influence on bone mass. Numerous studies have demonstrated that GH primarily acts through the release of insulin-like growth factor 1 (IGF-1) [72–74], which bind to glycoprotein-coupled receptors on osteoblasts, thereby promoting osteoblast proliferation [50, 73]. FSH, a major regulator of sex hormone secretion, is regulated by the hypothalamic secretion of gonadotropin-releasing hormone (GnRH). FSH directly affects bone cells and plays a role in promoting bone mass. FSH receptors are present in osteoblasts, their precursors, and MSCs, but not in mature osteoblasts [51, 52]. In vitro experiments have revealed that FSH binding to receptors on osteoblasts and their precursors stimulates osteoclast differentiation and function [53]. However, while the overall regulation of human bone mass by FSH has not been conclusively demonstrated in vitro experiments, some non-human studies suggest its negative impact on bone [51]. TSH, regulated by the hypothalamic secretion of thyrotropin-releasing hormone (TRH), affects bone metabolism through direct binding to TSH receptors expressed in osteoblasts and osteoclasts [54]. Additionally, TSH indirectly influences bone modeling via its effects on thyroid secretion. In osteoclasts, activation of TSH receptors inhibits signaling pathways involving Janus kinase (JNK) and nuclear factor kappa-B (NF-κB) inhibitor alpha (NFKBIA/Iκ-Bα), as well as the activities related to Jun proto-oncogene activator protein 1 (AP-1) transcription factor subunit (JUN/c-Jun) and NF-κB, and the expression of genes such as *AP-1* and tumor necrosis factor-α (*TNF-α*) [55]. Activation of TSH receptors in osteoblasts downregulates their differentiation and proliferation through regulators like Runt-related transcription factor 2 (RUNX2), osterix (OSX), and low-density lipoprotein receptor-related protein 5 (LRP5) [56]. Mice lacking TSH receptors in osteoblasts exhibit a phenotype characterized by low bone mass [54]. Similarly, regular administration of low-dose TSH injections to rats or mice increased bone mass [55]. ACTH, regulated directly by hypothalamic corticotropin-releasing factor (CRH), is released from the pituitary gland to stimulate glucocorticoid secretion from the adrenal gland. Glucocorticoids play a significant role in maintaining bone homeostasis [57]. Chronic elevation of glucocorticoid levels is a recognized cause of osteoporosis and reduced bone density [58]. Specifically, the binding of glucocorticoids to osteoblast receptors can directly inhibit osteoblast formation [59]. Additionally, glucocorticoids have been found to negatively impact growth plate cartilage proliferation [59].

Brain-derived EVs from patients with Alzheimer’s disease have been investigated for their role in both bone and brain physiology. Liu et al. [15] found that brain-derived miR-483-5p can effectively traverse the blood–brain barrier (BBB) to affect bone homeostasis. Specifically, miR-483-5p originating from Alzheimer’s disease brain was observed to induce a shift in the differentiation of bone marrow MSCs (BMSCs) from osteogenesis to adipogenesis, resulting in an imbalance between bone and fat tissue. In clinical settings, traumatic brain injury (TBI) has been shown to exacerbate bone healing processes [75]. However, the specific mechanism underlying this phenomenon is still under investigation. Xia et al. [76] demonstrated that hippocampal neurons release miR-328a-3p and miR-150-5p following TBI, which targeted osteoprogenitors to stimulate bone formation. They further injected a hydrogel enriched with miR-328a-3p into rat models with calvarial defects and observed a remarkable reparative effect. The brain-bone axis represents a dynamic system intricately regulated by many factors, each offering unique therapeutic targets for disorders related to bone. This axis serves as a nexus for potential interventions ranging from hormonal and neuropeptide pathways to neurotransmitter and CB signaling.

#### Bone to brain: bone-derived signals influencing neural function

A substantial body of experimental and clinical evidence demonstrates a reciprocal relationship between bone and the brain. Bone exerts its influence on the brain through various mediators derived from bone, including OCN, lipocalin 2 (LCN2), osteocyte-specific sclerostin (SOST), Dickkopf 1 (DKK1), and EVs (Fig. 2a, Table 1).

OCN, a mediator derived from bone, is induced by the bone gamma-carboxyglutamate protein expressed in osteoblasts to participate in systemic regulation [60]. OCN is implicated in brain development and cognition and has been shown to traverse the BBB into the CNS, promoting spatial learning and memory while preventing anxiety behaviors [61]. During fetal development, maternal OCN traverses the placenta to facilitate infant nervous system development and inhibit apoptosis of hippocampal neurons [61]. Furthermore, OCN is associated with neurodegenerative diseases and holds potential as a hormone for their treatment [61].

Similarly, LCN2, another glycoprotein derived from bone, is capable of crossing the BBB and binding to the melanocortin 4 receptor (MC4R) in the hypothalamus. This interaction plays a crucial role in mediating the anorexia pathway and regulating energy metabolism [62, 63]. In an MC4R-deficient mouse model, it was observed that there was a bias towards bone formation with increased bone mass in terms of maintaining bone homeostatic balance. Interestingly, despite correcting severe obesity which is typically associated with MC4R deficiency, the elevated levels of BMD were not restored [64].

Moreover, SOST, an osteocyte-specific glycoprotein, disrupts the Wnt/β-catenin signaling by binding with LRP4/5/6, leading to the disturbance of bone metabolic homeostasis in favor of bone resorption [65, 77]. Consequently, inhibiting SOST secretion has been proposed as a therapeutic approach for reduced bone density [66]. Romosozumab, a monoclonal antibody targeting SOST, has been developed and approved in the USA and Europe for the treatment of severe osteoporosis [78]. Within the brain, the Wnt/β-catenin pathway plays a crucial role in processes such as neurotransmission, neuronal metabolism, synaptic plasticity, and BBB function. The involvement of the Wnt/β-catenin pathway is also relevant to Alzheimer’s disease pathophysiology [79]. However, it should be noted that SOST is expressed in other tissues as well [67]. Therefore, further studies are needed to confirm whether osteocyte-derived SOST can influence Wnt/β-catenin signaling in the brain [68].

Furthermore, DKK1 functions as an antagonist of the Wnt signaling pathway. It exerts its antagonistic effects by binding to LRP6 in bone tissue, leading to a reduction in BMD when overexpressed [69, 80]. More importantly, DKK1 plays a crucial role in cell death and differentiation during embryonic development, with its overexpression resulting in neuronal dysfunction and apoptosis [70]. Additionally, DKK1 expression is generally upregulated in the aging brain [81], while Wnt pathway signaling is frequently diminished [71]. DKK1 serves as a marker commonly found in neurodegenerative diseases such as Alzheimer’s disease where it is upregulated among clinical patients [82].

The efficacy of BMSCs in the treatment of CNS diseases has been extensively researched and remains a topic of debate. Transplanted BMSCs has the potential to promote nerve repair by adopting functional neuronal and glial cell phenotypes [83–85]. However, it has been suggested that the primary mechanism through which BMSCs function is paracrine signaling rather than intracellular mechanisms [86]. EVs derived from BMSCs (BMSC-EVs) offer therapeutic potential due to their inherent stability, limited immunogenicity, and ability to cross the BBB or targeted delivery to the affected area [86]. These discoveries advance our understanding of BMSC-EVs and their contributions to brain protection and repair. By modulating microglial phenotypes from M1 to M2, BMSC-EVs alleviate cerebral infarction and mitigate short-term neurobehavioral impairments in rats [87].

The roles of non-coding RNAs within EVs have been investigated due to their capacity for transmitting information [88]. BMSC-EVs enriched with miR-124 were administered to mice in a transient middle cerebral artery occlusion (MCAO) model, resulting in reduced activation of proinflammatory microglial, preservation of BBB integrity, and mitigation of stroke-induced injury, potentially involving peroxiredoxin 1 [89]. While miR-124-3p is downregulated in rats with hypoxic-ischemic brain damage (HIBD), treatment with BMSC-EVs can upregulate miR-124-3p levels, leading to improved neurologic function, inhibition of oxidative stress, and reduction in neuronal apoptosis [90]. BMSC-EVs enriched with miR-124 hold promising therapeutic potential for the treatment of clinical HIBD.

LCN2 demonstrates elevated expression and functions as a downstream target of miR-138-5p. In their study utilizing an MCAO mouse model to investigate the in vivo impact of miR-138-5p carried by BMSC-EVs in ischemic stroke, Deng et al. [91] demonstrated that miR-138-5p attenuated neurological impairment in ischemic stroke through targeted regulation of LCN2, thereby promoting astrocyte proliferation and suppressing inflammatory responses. Additionally, using a rat MCAO model, Dong et al. [92] found that BMSC-EVs ameliorated cerebral infarction via transfer of miR-23a-3p. Moreover, Singleton et al. [93] revealed that BMSC-EVs activated NF-κB signaling and facilitated osteoclast differentiation from bone marrow cells in mice with TBI. These findings imply a potentially pivotal role for miR-1224 in inducing osteoclast differentiation during TBI-induced bone loss [93].

BMSC-EVs ameliorated HIBD in mice with MCAO by modulating the histone deacetylase 4 (HDAC4)/B-cell leukemia/lymphoma 2 (BCL2) axis and delivering miR-93 to prevent neuronal death in the hippocampus [94]. BMSC-EVs containing the long non-coding RNA ZNFX1 antisense RNA 1 (ZFAS1) exhibited a potentially therapeutic effect on ischemic brain injury [95]. Shen et al. [96] demonstrated that BMSC-EVs transported miR-410 to target HDAC4, thereby preventing brain damage in hypoxic-ischemic mice. The protective effect may be attributed to the downregulation of phosphatase. Xiong et al. [97] reported that miR-129-5p derived from BMSC-EVs alleviated early brain injury in rats after subarachnoid hemorrhage by inhibiting the antiapoptotic and anti-inflammatory effects mediated via the high mobility group box 1 (HMGB1)/Toll-like receptor 4 (TLR4) pathway. Additionally, Cui et al. [98] reported elevated levels of miR-21 in the brains of Alzheimer’s disease mice after treatment with EVs derived from hypoxia-preconditioned BMSCs. Moreover, supplementation with miR-21 reversed cognitive deficits and prevented degenerative features in APP/PS1 mice. Furthermore, Sha et al. [99] emphasized the critical role of the Wnt/β-catenin pathway activation and the delivery of miR-29c-3p to hippocampal neurons via BMSC-EVs for reducing amyloid-beta deposition and inflammatory cytokines thus ameliorating cognitive function in rats with Alzheimer’s disease.

In summary, the brain-bone axis is a dynamic system regulated by many signaling pathways. Factors influencing this axis can be categorized into neuropeptides (e.g., NPY and CART), neurotransmitters (e.g., 5-HT and DA), CBs, Semas, EVs, and pituitary hormones (e.g., GH and FSH). Neuropeptides such as NPY and CART directly impact osteoblast and osteoclast activity, thereby influencing bone formation and resorption. Neurotransmitters like 5-HT and DA exhibit varying effects on bone mass, often linked to their systemic levels in different diseases. CBs demonstrate a dual role in bone formation and resorption through their receptors. Semas including SEMA3A and SEMA4D regulate the activities of osteoblasts and osteoclasts, presenting potential therapeutic targets. Pituitary hormones regulated by the brain also significantly influence bone metabolism. Brain-derived EVs show promising roles in promoting both bone and brain health through specific microRNAs that affect bone homeostasis. In terms of clinical applications, these factors offer both advantages and challenges. Neuropeptides and neurotransmitters hold potential as therapeutic targets but require precise modulation due to their systemic effects. CB-based treatments offer promise but necessitate careful balancing to avoid adverse effects. Sema-targeted therapies could be effective but need further exploration for specificity. Pituitary hormone therapies have shown efficacy but require individualized treatment plans due to complex hormonal interactions. Brain-derived EVs represent a novel area with promising potential for targeted therapy with minimal invasiveness; however, their clinical translation is still in the early stages.

### Lung-bone axis

In the interplay between the lungs and bone, primary lung lesions such as lung tumors and chronic obstructive pulmonary disease (COPD), cystic fibrosis (CF), chronic coronavirus disease 2019 (COVID-19), and regional inflammation lesions, produce factors that exert effects on bone cells. Conversely, bone produces related factors that affect the lungs. Most studies on the cross-linking between the lungs and bone have primarily focused on lung diseases [100–104]. Their findings suggest that BMSC-EVs could represent a promising therapeutic strategy for acute lung injury (ALI) and other respiratory diseases. Therefore, this section aims to describe the interactions between the lungs and bone in relation to lung diseases (Fig. 2b).

In one study, it was observed that lung tumors stimulated the generation and recruitment of tumor-supportive BMSCs [100]. Specifically, these tumors activated osteoblasts residing in bone tissue which subsequently triggered the production of neutrophils. Engblom et al. [100] discovered that primary lung tumors in mice remotely activated osteoblasts expressing OCN^+^ by releasing soluble receptors for advanced glycation end products into circulation. The activation of osteoblasts resulted in generating a subset of neutrophils characterized by their elevated expression of sialic acid-binding Ig-like lectin F. Ultimately, it was found that these neutrophils returned to the site of the lung tumor, where they provided support for tumor growth.

COPD is recognized as a chronic inflammatory pulmonary disease accompanied by various intricate syndromes, including osteoporosis and diabetes, primarily induced by smoking. Osteoporosis-associated conditions can further exacerbate pulmonary function and symptoms in COPD patients, leading to a detrimental cycle. The mechanisms underlying osteoporosis in individuals with COPD remain unclear. During the initial stages of inflammation in COPD patients, exposure of lung epithelial cells to smoke particles results in the release of factors such as interleukin (IL)-1, TNF-α, and IL-8, contributing to the recruitment of immune cells. The post-inflammatory response also involves activation of T helper 1, 2, and 17 cells along with sustained activation of lung stem cells. Multiple factors produced during lung regional inflammation drive increased systemic inflammation and comorbidities (including osteoporosis), some of which are also considered therapeutic targets for stage 3 COPD [101, 105–107].

The most prevalent autosomal recessive genetic disorder, CF, is caused by mutations at a specific locus within the CF transmembrane conductance regulator gene [108]. Clinically, CF is accompanied by osteopenia and osteoporosis [109]. Patients with CF have been found to exhibit reduced levels of circulating OCN associated with vertebral fractures [110]. Conversely, lower serum OCN levels were correlated with higher ratio of RANKL/osteoprotegerin (OPG) in children aged 5 – 9 years with CF, indicating an imbalance favoring bone resorption in terms of bone homeostasis [102].

COVID-19 is a syndrome characterized by pneumonia and other complications resulting from infection with severe acute respiratory syndrome coronavirus 2 (SARS-CoV-2) [103]. Qiao et al. [111] used SARS-CoV-2-infected hamsters as a model for human infection to study bone metabolism during the acute pneumonia and recovery phases. They observed an upregulation of tartrate-resistant acid phosphatase (TRAP)^+^ osteoclast expression of nuclear factor of activated T cells 1 (*Nfatc1*), which was associated with a notable increase in bone resorption. More importantly, analysis of various bone tissues collected from hamsters revealed systemic rather than localized bone loss. Such bone loss in patients with COVID-19 may impede recovery, as low BMD is a risk factor for vertebral fractures and likely contributes to the deterioration of respiratory function during the rehabilitation phase. In addition, they also found that hamsters infected with SARS-CoV-2 exhibited an immune response in the respiratory tract, leading to the production of inflammatory cytokines (i.e., IL-1β, TNF-α, and interferon γ). These cytokines were transported through the bloodstream to skeletal tissue where they promoted osteoclastogenesis.

The bone is a frequently affected site for the metastasis of lung cancer. Research findings have demonstrated that EVs in lung cancer possess the ability to influence bone physiology, as depicted in Fig. 2b. Taverna et al. [112] observed that EVs derived from non-small cell lung cancer (NSCLC) can activate the amphiregulin-induced epidermal growth factor receptor (EGFR) pathway, resulting in increased *RANKL* expression in bone cells. Consequently, RANKL stimulates the production of proteolytic enzymes indispensable for osteoclastogenesis, thereby initiating a detrimental cycle associated with osteolytic bone metastasis. Additionally, Xu et al. [113] demonstrated that exosomal miR-21 derived from the tumor tissues of patients with lung adenocarcinoma promotes osteoclast formation, making it a promising therapeutic target for managing bone metastasis.

Furthermore, studies have revealed that BMSC-EVs provide novel insights into the treatment of bone diseases. Hua et al. [114] investigated the regulatory mechanisms and potential therapeutic applications of small BMSC-EVs containing miR-34c in targeting the epithelial sodium channel (ENaC) via myristoylated alanine-rich protein kinase C substrate (MARCKS). Their study using a mouse model demonstrated that miR-34c could enhance ENaC expression by binding with MARCKS and activating the phosphoinositide 3-kinase (PI3K)/protein kinase B (Akt) pathway, thereby promoting alveolar fluid clearance and potentially alleviating lung edema. Liu et al. [115] explored the role of miR-let-7i encapsulated in BMSC-EVs in lung cancer progression. They discovered that miR-let-7i could suppress lung cancer through the lysine demethylase 3A (KDM3A)/doublecortin-like kinase 1 (DCLK1)/FXYD domain-containing ion transport regulator 3 (FXYD3) axis in vivo using nude mice, suggesting a potential therapeutic strategy for treating lung cancer. Additionally, Liu et al. [116] investigated how hypoxic BMSC-EVs promote lung cancer progression and elucidated the involvement of miR-328-3p and the neurofibromin 2 (NF2)-mediated Hippo axis in lung cancer development. They revealed that under hypoxic conditions, BMSC-EVs stimulated the expansion and dissemination of lung cancer cells through miR-328-3p- and NF2-mediated Hippo pathways in vivo using nude mice models. Wang et al. [117] proposed the potential role of microRNA transfer in mediating the effects of MSC-EVs on ALI. Their findings suggest that the transfer of lentivirus-transduced BMSCs carrying miR-27a-3p or knocking out miR-27a-3p in vivo can modulate macrophage polarization and alleviate ALI. Liang et al. [118] investigated the inhibitory effects of miR-144 derived from BMSC-EVs on NSCLC progression by targeting cyclins E1 and E2, which was further validated in vivo experiments.

In summary, the lung-bone axis entails an intricate interplay of cellular and molecular mechanisms. The emerging role of BMSC-EVs is particularly noteworthy, offering innovative therapeutic strategies for ALI and other respiratory conditions. Given the nascent state of current literature, numerous mechanistic details remain to be elucidated. For example, the precise involvement of cytokines such as IL-1 and TNF-α in COPD-induced osteoporosis remains unclear, necessitating further investigation. Similarly, the systemic bone loss observed in patients with COVID-19 raises new questions regarding long-term rehabilitation and treatment strategies. These gaps in our understanding not only offer avenues for future research but also represent potential targets for therapy. From a clinical perspective, these interactions present both opportunities and challenges. Understanding these bidirectional influences can assist in developing targeted therapies for conditions such as lung cancer metastasis and ALI. However, the complexity of these interactions poses a challenge specifically when ensuring focused treatment without unintended systemic effects.

### Liver-bone axis

The interaction between the liver and bone differs under normal physiological and pathological conditions (Fig. 2c). Patients with chronic liver disease often experience osteoporosis/osteopenia/fractures, particularly those with cirrhosis and cholestatic diseases. However, the mechanisms underlying osteoporosis in individuals with chronic liver disease are multifactorial, and the precise mechanisms remain unclear. Thioacetamide can induce liver damage in Sprague–Dawley rats, which serve as ideal animal models for studying abnormal bone metabolism after liver injury [119].

Extensive in vitro and in vivo evidence has shown that IL-6 is upregulated during liver injury and subsequently affects bone metabolism in various liver diseases [120, 121]. Additionally, both animal model studies and clinical data indicate that ethanol appears to stimulate osteoclast activity by inducing IL-6 and TNF-α [122, 123], which have similar effects on osteoporosis observed in viral hepatitis and nonalcoholic steatohepatitis. Moreover, vitamin D and 25-hydroxylation of vitamin D levels were found to be decreased in patients with nonalcoholic fatty liver disease [124] or alcoholic liver disease [125], characterized by impaired hepatic function and cholestasis. These reductions were associated with the presence of osteoporosis [126, 127].

Fibroblast growth factor-21 (FGF-21) is a hepatic-produced factor that, when deficient, can impair liver cell function [128]. FGF-21 exerts a negative impact on bone mass [129]. Overexpression of FGF-21 in transgenic mice resulted in a significant decrease in BMD [130]. Bone morphogenetic protein-9 (BMP-9), a circulating factor produced by hepatic stellate cells, is expressed in the adult liver [131]. Zhou et al. [132] demonstrated that in ovariectomized mice, the overexpression of BMP-9 not only led to increased levels of BMP-9 in liver but also inhibited bone resorption activity, enhanced bone formation, and improved bone strength. Additionally, Guanabens et al. [133] suggested that SOST plays a pivotal role in regulating the Wnt/β-catenin pathway in bone formation among patients with primary biliary cholangitis. SOST is produced by osteocytes and inhibits osteoblast proliferation and differentiation. It binds to LRP5/6 to counteract Wnt signaling and restricts bone formation [134, 135]. Importantly, hepatocyte-secreted IGF-1 has an anabolic effect on bone growth by inhibiting osteoblast apoptosis and promoting osteoblastogenesis through stabilization of the Wnt/β-catenin pathway [136, 137]. Furthermore, IGF-1 reduced bone resorption through the OPG and RANKL signaling pathways [138].

Hepatic osteodystrophy (HOD) is a chronic liver disease characterized by metabolic bone disease, often presenting with significant bone loss. Lu et al. [139] found that upregulating hepatic phosphatase protein phosphatase 2 catalytic subunit alpha (Ppp2ca/Pp2aca) during HOD resulted in the downregulation of the hepatokine lecithin-cholesterol acyltransferase (LCAT) activity. LCAT dysfunction further exacerbated bone loss in mice with HOD. The underlying mechanism involves LCAT’s role in maintaining intracellular cholesterol dynamic homeostasis, which supports the activities of osteoblasts and osteoclasts. Interestingly, upregulation of LCAT expression in HOD model mice improved liver fibrosis and bone loss phenotypes.

Bidirectional communication mediated by EVs exists between liver cancer and bone (Fig. 2c). Wei et al. [140] transfected HepG2 cells (a hepatoma cell line) with miR-181d-5p mimics and inhibitors, followed by isolated EVs for coculture with BMSCs to evaluate their growth potential. Subsequently, the cocultured BMSCs were transplanted into nude mice to observe the progression of liver cancer cells. The study demonstrated that HepG2-EVs induced BMSC differentiation and exacerbated liver cancer metastasis through the delivery of miR-181d-5p both in vitro and in vivo. Han et al. [141] revealed that miR-3190-5p is transferred from EVs derived from bone-metastasized hepatocellular carcinoma to orthotopic tumor cells, thereby enhancing their metastatic capacity. This effect was achieved by downregulating AlkB homolog 5 RNA demethylase (ALKBH5) expression, which modulates gene expression through N^6^-methyladenosine-dependent and -independent manners.

BM-derived EVs have long been transplanted via orthotopic liver transplantation (OLT) in mice, which is a focal point of research due to its potential protective and reparative effects in liver diseases. Using an OLT mouse model, Zhou et al. [142] discovered that BMSC-EVs could induce M2 polarization of Kupffer cells and carry miR-22-3p, thereby improving liver function and reducing inflammatory cytokines. Haga et al. [143] explored the potential of BMSC-EVs in enhancing survival in a mouse model of lethal hepatic failure. Their results showed that deficiency of Y-RNA-1 attenuated the mitigating effects of BMSC-EVs on hepatocyte apoptosis induced by TNF-α/actinomycin D in vitro, suggesting a therapeutic role of Y-RNA-1 in repairing liver injury and highlighting the value of clinical studies [143]. Xuan et al. [144] revealed that enriching miR-148a-5p in BMSC-EVs could alleviate thioacetamide-induced hepatic fibrosis in mice by targeting SMAD family member 4. Zhang et al. [145] used a mouse model of acute liver failure to explore the molecular mechanisms regulating hepatocyte proliferation and apoptosis in response to treatment with human BMSC-EVs. Specifically, they found that miR-20a-5p derived from BMSCs promoted hepatocyte proliferation and inhibited apoptosis through the phosphatase and tensin homolog (PTEN)/Akt pathway by downregulating PTEN expression and promoting Akt as well as glycogen synthase kinase-3 β phosphorylation.

The section delved into the intricate interplay between the liver and bone, distinguishing factors based on their roles in the liver-bone axis and evaluating their clinical implications. Factors influencing this crosstalk were classified as follows: 1) inflammatory mediators (e.g., IL-6 and TNF-α), which are upregulated during liver injury and impact bone metabolism; 2) metabolic regulators (e.g., vitamin D and FGF-21), with vitamin D deficiency associated with osteoporosis and FGF-21 negatively affecting bone mass; 3) regulators of bone formation and resorption (e.g., BMP-9, SOST, and IGF-1), where BMP-9 enhances bone formation, SOST restricts it, and IGF-1 promotes osteoblastogenesis; and 4) EVs, which play roles in interactions between liver cancer cells and bone, offering potential for therapeutic applications. The advantages for clinical application include potential therapeutic targets such as the Wnt/β-catenin pathway and BMSC-EVs for liver repair. However, limitations exist due to an incomplete understanding of molecular pathways, particularly regarding TNF-α and IL-6’s relation to osteoporosis, as well as the unclear role of IGF-1 in liver disease-related bone metabolism.

### Heart-bone axis

Data from the World Health Organization indicates that cardiovascular diseases (CVDs), encompassing conditions such as atherosclerosis, myocardial obstruction, and ischemic heart disease, are the leading cause of mortality globally. Circulating endothelial progenitor stem cells (EPCs) derived from bone marrow have a complex bidirectional relationship with CVD (Fig. 2d). There is evidence suggesting that EPC markers, including TNF receptor superfamily member 4 (TNFRSF4/CD134), CD34 molecule (CD34), and vascular endothelial growth factor receptor [146], not only serve as indicators for CVD but also promote myocardial repair in the presence of ischemic injury [147]. Similarly, Rohde et al. [148] recently proposed that the cardiovascular system also impacts bone marrow.

Regarding the effects of bone on the cardiovascular system, Werner et al. [149] conducted a study involving 519 patients with coronary artery disease and predicted adverse CVD based on the number of circulating CD34^+^ EPCs. Dimmeler et al. [150] suggested that the activation of the early apoptosis pathway in EPCs could signify the initial stage in the formation of atherosclerotic lesions and the development of heart failure in atherogenic arteries. These observations indicate a strong association between CVD and EPCs derived from bone marrow.

In terms of the interaction between the cardiovascular system and bone marrow, Rohde et al. [148] investigated how vascular endothelial cells in bone marrow relate to hematopoiesis in individuals with CVD. The researchers discovered an elevated level of hematopoiesis among individuals with hypertension, recent acute myocardial infarction, or both hypertension and atherosclerosis compared to healthy control individuals. Furthermore, there was a specific increase observed in immune cells derived from a subset of hematopoietic stem and progenitor cells known as myeloid progenitor cells.

The transplantation of BMSC-EVs into infarcted myocardium has emerged as a prominent area of research. Transplanting BMSCs into the heart results in the release of EVs that play a crucial role in cardioprotection and repair (Fig. 2d). Yu et al. [151] investigated the cardioprotective effects of EVs derived from BMSCs overexpressing GATA binding protein 4 in rats with myocardial infarction, revealing that treatment with these EV injections reduced myocardial infarct size and increased miR-19a and miR-451 expression in cardiomyocytes. Feng et al. [152] found that miR-22, enriched in EVs secreted by MSCs, transferred to preconditioned rats cardiomyocytes experiencing cardiac ischemia, effectively mitigated ischemia-induced cardiomyocyte apoptosis by directly targeting methyl CpG-binding protein 2.

Additionally, Sun et al. [13] found an association between high circ-G042080 expression observed in several patients with primary myeloma-associated myocardial injury and reduced ventricular ejection fraction and systolic blood pressure, which are hallmarks of myocardial injury. This study suggests the potential of EVs mediating bone-cardiac communication. Moreover, Cheng et al. [153] investigated the role of mouse myocardial microRNAs in mediating functional crosstalk between the ischemic heart and bone marrow via circulating EVs, exploring their potential impact on cardiac repair. They revealed that after acute myocardial infarction, circulating EVs predominantly carry specific myocardial microRNAs such as miR-499, miR-133, miR-1, and miR-208 which are selectively imported into peripheral organs particularly the bone marrow, where they downregulate gene C-X-C motif chemokine receptor 4 (*CXCR4*) expression while increasing the number of circulating progenitor cells.

In light of the existing literature, the heart-bone axis emerges as a complex yet underexplored domain with profound implications for cardiovascular and bone health. The factors can be categorized into three groups: 1) bone marrow-derived EPCs, which serve as markers and agents of myocardial repair in CVD, with CD34^+^ EPCs being predictive of adverse CVD outcomes; 2) vascular endothelial cells in the bone marrow, which influence hematopoiesis and myeloid cell production in CVD conditions; and 3) bone marrow-derived EVs, which play a role in cardiac repair through the transfer of microRNAs and other molecules. The advantages for clinical applications include the potential use of EPCs and bone marrow-derived EVs in myocardial repair, as well as the use of cellular markers for predicting and managing CVD. However, there are also disadvantages such as the complex and incompletely understood interaction mechanisms between the heart and the bone marrow, along with variability in patient responses. Therefore, we call for a more integrated approach to studying the heart-bone axis by incorporating advanced molecular techniques and multi-omics analyses. We also highlight the potential therapeutic targets such as the modulation of specific microRNAs or using EVs for targeted drug delivery. This summary underscores the intricate bidirectional relationship between the heart and bone while emphasizing how bone marrow-derived cells and EVs hold promise in treating CVD.

### Kidney-bone axis

#### Kidney to bone

Renal effects on the bone are now generally focused on mineral and bone disease (MBD) resulting from chronic kidney disease (CKD). MBD in CKD is a physiological imbalance of calcium, phosphate, and parathyroid hormone (PTH) due to abnormal renal metabolism (Fig. 2e). Specifically, as glomerular filtration performance decreases, there is a reduction in renal tubule reabsorption levels and phosphate reabsorption, resulting in elevated serum phosphate concentrations [154]. Increased phosphate levels stimulate osteoblasts to secrete FGF-23, thereby increasing PTH levels [155–157]. Clinically, hypocalcemia is commonly observed in CKD patients, which further promotes PTH hyperactivity [157]. A recent study also suggests that the increased secretion of FGF-23 by osteocytes is influenced by the elevation of glycerol-3-phosphate in CKD [158]. FGF-23 also inhibits the active form of vitamin D, subsequently impacting PTH elevation [159]. Eventually, the elevations in FGF-23 and PTH aggravate bone resorption and disrupt bone homeostasis resulting in MBD [160, 161]. In cases of kidney injury, activin A is upregulated in the blood, positively affecting renal cell fibrosis [162]. In mouse models, inhibition of activin A secretion contributes to kidney injury repair [163]. In bone, activin A is secreted by osteolineage cells and abundantly presents in the extracellular matrix (ECM). Although it has multiple effects on bone physiology involving various cell types, it is generally considered a negative regulator of bone formation [164].

#### Bone to kidney

The bone mainly affects the kidney through FGF-23, which inhibits phosphate reabsorption in renal tubules by suppressing the expression of renal phosphate transporters within renal proximal tubules [165]. Additionally, FGF-23 hinders the expression of 1-alpha hydroxylase in the renal proximal tubule while stimulating 24-hydroxylase. This enzyme converts 25-OH vitamin D and calcitriol into inactive substances [165, 166]. Moreover, a recent study identified immature myeloid cells in the bone marrow as a significant cellular source of soluble plasminogen activator, urokinase receptor, a circulating biomarker of inflammation that significantly impacts kidney disease pathogenesis [167].

In preclinical and clinical studies, BMSCs have been confirmed to possess substantial therapeutic effects on kidney diseases such as acute kidney injury (AKI) and CKD (Fig. 2e). Shen et al. [168] investigated the potential therapeutic role of C–C motif chemokine receptor 2 (CCR2) in BMSC-EVs concerning ischemia/reperfusion kidney injury. They demonstrated that CCR2 has a high affinity for its ligand, C–C motif chemokine ligand 2 (CCL2), and that CCR2-positive EVs could decrease free CCL2 levels, inhibit its recruitment or activation of macrophages, and significantly impact inflammatory regulation and renal injury repair. Mice with renal ischemia–reperfusion injury were administered *CCR2* knockout BMSC-EVs resulting in impaired reparative effects.

Cao et al. [169] demonstrated the therapeutic effect of miR-200a-3p in BMSC-EVs on acute kidney ischemia–reperfusion injury and its underlying mechanism. Specifically, in the AKI model mice, miR-200a-3p downregulated kelch-like ECH-associated protein 1 expression and inhibited the upregulation of nuclear factor erythroid 2-related factor 2 and superoxide dismutase 2 while regulating mitochondria to exert antioxidant effects for renal damage repair. Tapparo et al. [170] utilized microRNA-enriched EVs from different species to treat an AKI model by assessing renal function and morphology. They performed bioinformatic analysis and identified that miR-127-3p, miR-29a-3p, miR-10a-5p, and miR-486-5p as crucial factors in renal injury repair. Among them, miR-486-5p was found to be the most abundant microRNA in the exosomal fraction, demonstrating its superior efficacy in promoting AKI recovery.

Bruno et al. [171] discovered that BMSC-EVs carry specific mRNAs [cyclin B1 (*CCNB1*), cyclin-dependent kinase 8 (*CDK8*), and cell division cycle 6 (*CDC6*)], which contribute to their pro-proliferative effect on mouse renal tubular epithelial cells both in vitro and in vivo. Wang et al. [172] reported the successful delivery of miR-let7c to damaged kidneys in mice with unilateral ureteral obstruction using engineered BMSCs. The therapy with miR-let7c reduced kidney injury by downregulating the expression levels of collagen type IV alpha 1 (*Col4a1*), matrix metalloproteinase-9 (*Mmp-9*), transforming growth factor (TGF) beta 1 (*Tgfb1*), and TGF beta receptor 1 (*Tgfbr1*) in the kidneys of mice with unilateral ureteral obstruction.

The kidney-bone axis represents a compelling paradigm of endocrine interplay, particularly in the context of CKD and MBD. While the role of FGF-23 and PTH in modulating bone and renal physiology is well documented, the precise mechanisms and potential therapeutic targets remain an active area of research. For example, recent findings on the role of glycerol-3-phosphate in elevating FGF-23 levels in CKD offer a new avenue for intervention, but further validation is needed. Similarly, the therapeutic potential of BMSC-EVs in renal diseases is promising but not yet universally accepted due to the heterogeneity in study designs and outcomes. The clinical importance of understanding the kidney-bone axis cannot be overstated, especially given the high prevalence of CKD and associated comorbidities such as MBD. The potential for targeted therapies, such as modulating specific microRNAs or using EVs for drug delivery, offers tantalizing prospects for personalized medicine. However, these therapeutic avenues also necessitate a more nuanced understanding of the underlying molecular mechanisms, possibly through multi-omics approaches and machine learning algorithms for data integration.

### Muscle-bone axis

#### Muscle to bone

**Fig. 3:**
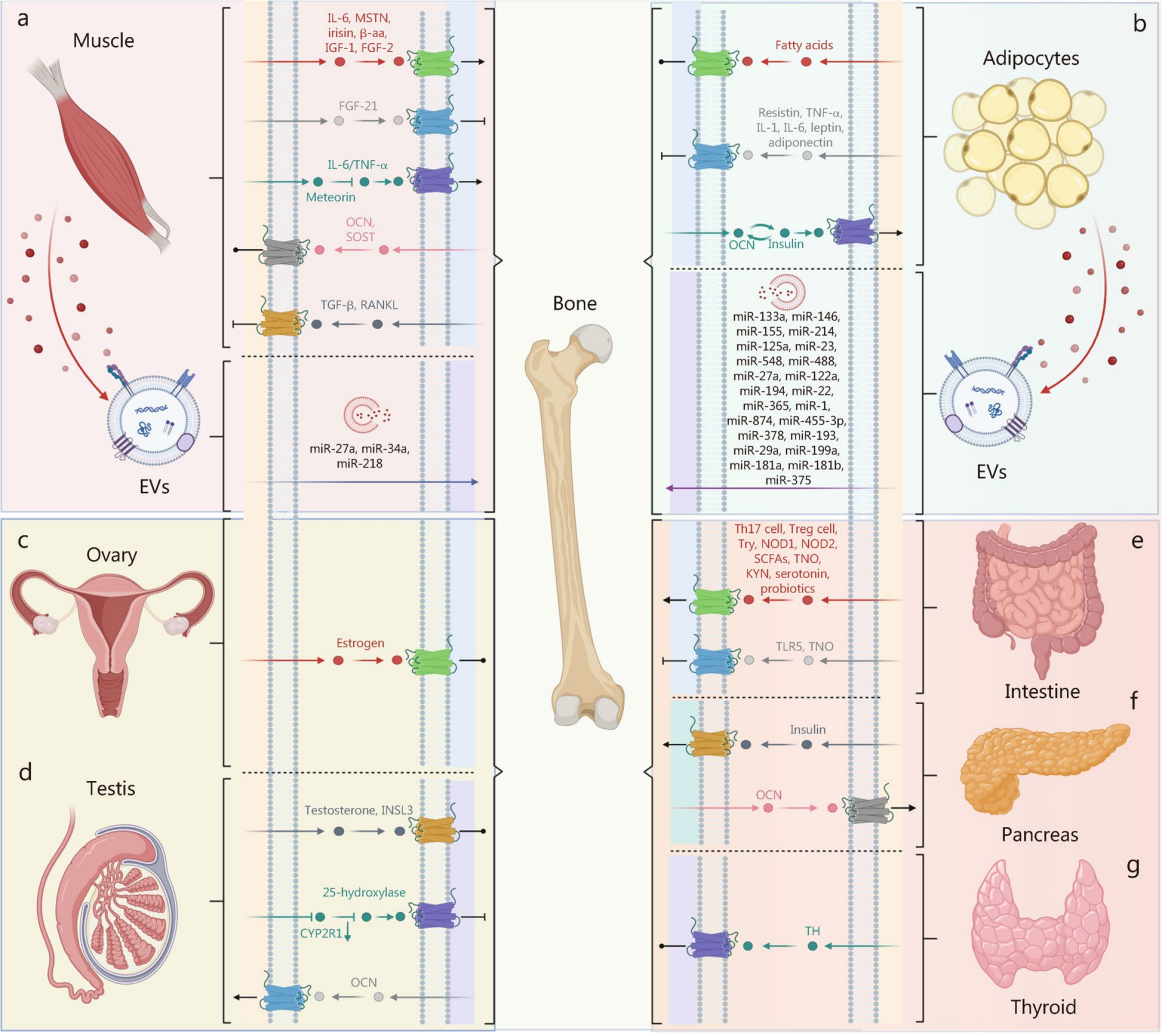
The systemic integration of bone-organ signaling axes. This figure maps the multidirectional signaling networks that connect bone with muscle, adipose tissue, gonads, intestine, pancreas, and thyroid, focusing on the molecular mediators involved in these interactions. **a** The bone-muscle axis is characterized by a reciprocal regulatory network, where cytokines, growth factors, and EVs such as IL-6, MSTN, and miR-218 influence myogenesis and osteogenesis, illustrating the integral role of muscle in bone homeostasis. **b** The bone-adipocyte axis delves into the metabolic crosstalk through adipokines and osteokines, highlighting the dual role of fatty acids and hormones such as leptin in energy metabolism and bone remodeling. The bone-ovary (**c**) and bone-testis (**d**) axes detail the endocrine interplay, with estrogen and testosterone being pivotal regulators of bone density and gonadal function, underscoring the importance of sex hormones in skeletal integrity. **e** The bone-intestine axis emphasizes the regulatory roles of intestine-derived immune signals, microbiota byproducts, and probiotics on bone health. **f** The bone-pancreas axis highlights the insulin-OCN feedback loop essential for energy metabolism and bone density regulation. **g** The bone-thyroid axis demonstrates the thyroid hormones’ direct impact on bone growth and metabolism. Collectively, these axes not only dictate the physiological regulation of bone density and turnover but also highlight the systemic implications of bone as a central organ in orchestrating overall homeostasis. IL-1/6 interleukin-1/6, MSTN myostatin, β-aa β-aminoisobutyric acid, IGF-1 insulin-like growth factor-1, FGF-21 fibroblast growth factor-21, TNF-α tumor necrosis factor-α, OCN osteocalcin, SOST sclerostin, TGF-β transforming growth factor-β, RANKL receptor activator of nuclear factor kappa-B ligand, INSL3 insulin-like 3, CYP2R1 cytochrome P450 family 2 subfamily R member 1, Th17 T helper 17 cells 17, NOD1/2 nucleotide-binding oligomerization domain 1/2, SCFAs short-chain fatty acids, TNO trimethylamine-N-oxide, Try tryptophan, KYN kynurenine, TLR5 Toll-like receptor 5, TH thyroid hormone

In the context of inflammation and aging, elevated secretion of IL-6 from skeletal muscle exerts its effects on bone through the circulatory system, leading to the induction of osteoclast formation [173]. Myostatin (MSTN), a protein secreted by muscle cells, stimulates RANKL expression to enhance osteoclast activity [174]. Regarding bone metabolism, FGF-21, an actin associated with glucose and lipid metabolism, inhibits osteocyte proliferation and bone marrow adipogenesis [130] (Fig. 3a).

Irisin enhances osteoblast activity by triggering the activation of transcription factor 4, thereby positively influencing bone health [175, 176]. Another myokine called β-aminoisobutyric acid (β-aa), is elevated during exercise and acts as an antioxidant to safeguard osteocytes against oxidative stress to prevent bone resorption [177]. Meteorin-like, another myo-factor, inhibits bone resorption by attenuating pro-inflammatory cytokines such as IL-6 and TNF-α. Furthermore, IGF-1 and FGF-2 are both growth factors produced by skeletal muscle, promoting osteoblast formation and thus enhancing overall bone metabolism [178, 179] (Fig. 3a).

Muscle-derived EVs have been shown to promote osteogenic differentiation in MC3T3-E1 cells through the β-catenin signaling pathway to the bone, as demonstrated by Xu et al. [180]. Specifically, EVs derived from C2C12 myoblasts or myotubes were found to enhance this process, with miR-27a identified as the primary functional factor. Furthermore, miR-27a has been implicated in enhancing myoblast differentiation [181, 182], and its mimic has shown potential in alleviating the muscle atrophy induced by CKD [183]. These findings collectively suggest that miR-27a may play a crucial role in mediating the communication between muscle-derived EVs and bone.

Recently, a fascinating study showed that miR-34a is significantly upregulated in skeletal muscle and serum EVs from aged mice. Fulzele et al. [184] reported an age-related increase in miR-34a levels in EVs positive for sarcoglycan alpha or derived from oxidatively stressed C2C12 cells. Importantly, these EVs were found to migrate to the bone marrow, where they induced BMSC senescence by suppressing the expression of sirtuin 1 (*Sirt1*). The elevated levels of miR-34a in aging muscle may contribute to muscle metabolic dysfunction, while reduced SIRT1 activity in aging muscle was associated with impaired muscle performance [185, 186].

Furthermore, MSTN was observed to suppress osteocyte-derived EVs containing miR-218, which were subsequently internalized by osteoblasts and hindered their differentiation [174]. MSTN also decreased the levels of miR-218 in EVs derived from both osteocytes and their parent cells. Notably, miR-218 was found to enhance the Wnt pathway by inhibiting *SOFT* expression. Specifically, EVs released by Ocy454 osteocytes treated with MSTN could be internalized by osteoblast and osteoclast precursor cells in vitro. These EVs then inhibited osteoblastic differentiation through modulation of the Wnt pathway, although they did not influence osteoclastic differentiation [174]. It is important to note that these conclusions are based on findings obtained through in vitro experiments and further studies conducted in vivo are warranted.

#### Bone to muscle

In this section, the effects of muscle on bone are described in terms of OCN, SOST, prostaglandin E_2_ (PGE_2_), TGF-β, RANKL, and EVs (Fig. 3a). OCN expression levels have been reported to increase during exercise but decline with age [187]. OCN secreted by end-stage osteoclasts can interact with the muscle receptor G protein-coupled receptor family C group 6 member A (GPCR6) to regulate muscle function [188]. Under pathological conditions, TGF-β released by the bone matrix reduces muscle strength by decreasing Ca^2+^ levels [189]. Moreover, OCN promotes IL-6 expression, which subsequently stimulates OCN expression in bone, positively affecting muscle growth. SOST secreted by osteocytes impedes bone formation through the canonical Wnt/β-catenin pathway [190]. Deficiency of SOST induced by *SOST* gene deletion or administration of SOST antibodies not only increases bone mass [191] but may also lead to sclerosteosis development [192]. Conversely, SOST expression decreases after bone or muscle loading [193, 194].

Furthermore, PGE2 is vital in enhancing the functionality of skeletal muscle-specific stem cells, leading to improved regeneration and strength through its interaction with the prostaglandin E receptor 4 (PTGER4) [195]. Additionally, PGE2 activated the β-catenin pathway by stimulating PI3K in osteocytes [196], and it promoted the proliferation of C2C12 myoblasts [197]. The signaling of PGE2 mitigates muscle atrophy and revitalizes muscle function. Therefore, targeting the PGE2-degrading enzyme 15-prostaglandin dehydrogenase could be a promising therapeutic strategy for preventing sarcopenia [198].

Produced by the bone matrix, TGF-β is another regulatory factor that significantly influences muscle growth. Treatment with TGF-β reduced specific muscle force but did not affect muscle mass in mice [199]. Moreover, TGF-β induced muscle weakness in mice with osteolytic cancer by elevating oxidative stress and causing calcium mishandling within muscles [189, 200]. RANKL also plays a pivotal role in mediating osteoclast formation, function, and survival [201]. Excessive expression of RANKL leads to bone loss and reduced muscle performance. Deletion of the RANKL receptor in muscles prevents denervation-induced muscle atrophy and dysfunction [202]. Treatment with anti-RANKL agents such as denosumab protects against skeletal muscle dysfunction while enhancing bone mechanical properties [203]. Notably, denosumab substantially augmented appendicular lean mass and enhanced handgrip strength, implying a close relationship between bone and muscle while suggesting denosumab as a potential innovative treatment for sarcopenia [204].

The muscle-bone axis is a critical interface for the interplay of various signaling molecules, growth factors, and EVs, orchestrating a complex regulatory network that profoundly impacts musculoskeletal health and disease. While the inhibitory roles of IL-6, MSTN, and FGF-21 in bone formation are well-established, emerging data on the beneficial effects of irisin and β-aa present new avenues for therapeutic interventions. The modulation of this axis by EVs, particularly those containing miR-27a and miR-34a, is an expanding field that necessitates further investigation. Understanding the clinical relevance of the muscle-bone axis is paramount, especially in the aging population where sarcopenia and osteoporosis often coexist. Targeted therapies, such as anti-RANKL treatment and modulating PGE2 signaling hold potential but require rigorous clinical validation. Furthermore, exploring the role of EVs in this axis could revolutionize our understanding and offer novel therapeutic strategies. However, it is crucial to acknowledge that most of the current knowledge stems from in vitro studies and animal models, thus translational research is imperative to validate these findings in humans.

### Adipocyte-bone axis

#### Adipose tissue to bone

Adipose tissue exerts its influence on bone through four mechanisms. Firstly, adipocytes release endocrine cytokines and growth factors that impact the function of osteoblasts and osteoclasts. Secondly, adipokines such as leptin and adiponectin regulate the sympathetic outflow from the CNS. Thirdly, paracrine factors produced by adipocytes within the bone marrow microenvironment affect neighboring cells in trabecular bone. Lastly, EVs derived from adipose tissue play a role in modulating bone physiology, pathology, and disease treatment differentially (Fig. 3b).

Regarding the endocrine influence of adipose tissue, visceral fat depots characterized by inflammation release cytokines such as resistin, TNF-α, IL-1, and IL-6. These cytokines disrupt bone remodeling by promoting bone resorption or inhibiting bone formation [205]. Similarly, both adiponectin and leptin have the potential to affect bone remodeling either through endocrine actions or by influencing the hypothalamic centers that regulate sympathetic activity [206–208]. The release of sympathetic impulses impedes osteoblast differentiation and promotes osteoclast recruitment, leading to the uncoupling of the bone remodeling unit.

Concerning the paracrine effects of adipose tissue, bone marrow adipocytes, which were initially discovered more than a century ago, constitute an essential component of the bone marrow environment and play a crucial role in both skeletal function and hematopoiesis [209]. Moreover, osteoblasts and adipocytes originate from common precursor MSCs, making their differentiation pathways closely interconnected [210]. The differentiation process of MSCs into either adipocytes or osteoblasts is highly regulated and involves various lineage-specific transcription factors. For example, RUNX2 and OSX are pivotal in promoting osteoblast differentiation, while peroxisome proliferator-activated receptor gamma (PPARG) plays a critical role in driving adipocyte differentiation [211]. Notably, inhibiting PPARG promotes osteogenesis while increasing PPARG activity reduces it, indicating a potentially mutually exclusive relationship between these two processes [212]. Additionally, the elevated production of adipose-related factors such as fatty acids can exert both positive and negative effects on metabolism within the bone marrow depending on the specific fatty acid involved and the type of receptor activation on BMSCs [213].

Adipose tissue-derived EVs play an important role in bone remodeling by regulating the function of osteoclasts and osteoblasts. Table 2 lists the microRNAs associated with obesity and osteoporosis as potential targets [214]. Interestingly, the release of EVs from white adipose tissue increases while that from brown adipose tissue decreases in osteoporosis. Furthermore, adipose tissue-derived EVs have been implicated as pivotal drivers of multiple myeloma (MM) tumorigenesis and disease progression, particularly through miR-181a and miR-181b which are also linked to adipose tissue and BMSCs [215]. Therefore, the exchange of EVs between bone marrow adipose tissue and MM cells mediated by these microRNAs [16] represents a promising mechanism supporting the progression of MM. Moreover, studies have found that adipose tissue-derived EVs regulate osteogenic differentiation and promote bone regeneration in vivo. Chen et al. [216] used miR-375-enriched stem cells derived from adipose tissue to improve the osteogenic differentiation of human BMSCs and new bone formation in a rat model.

**Table 2:** Target microRNAs associated with obesity and osteoporosis

EVs	Expression in OP patients	Expression in obesity patients	Function
White adipose tissue-derived EVs			
miR-133a	↑	↓	Promotes osteoclast differentiation and inhibits osteoblast differentiation
miR-146	**↑**	**↑**	Inhibits osteogenesis
miR-155	**↑**	**↑**	Inhibits osteogenesis
miR-214	**↑**	**↑**	Inhibits osteogenesis
miR-125a	**↑**	**↑**	Promotes osteoclast differentiation
miR-23	**↑**	**↑**	Inhibits osteogenesis
miR-548	**↑**	**↑**	Unknown
miR-488	**↑**	**↑**	Inhibits osteogenesis
miR-27a	**↑**	**↑**	Inhibits osteogenesis
miR-122a	**↑**	**↑**	Inhibits osteogenesis
miR-194	**↑**	**↑**	Inhibits osteogenesis
miR-22	**↑**	**↑**	Inhibits osteogenesis
Brown adipose tissue-derived EVs			
miR-365	**↓**		Ameliorates dexamethasone-induced suppression of osteogenesis
miR-1	**↓**		Stimulates osteogenesis and inhibits adipogenesis
miR-874	**↓**		Promotes the proliferation and differentiation of osteoblasts
miR-455-3p	**↓**		Protects osteoblasts from oxidative stress
miR-378	**↓**		Inhibits osteoclastogenesis
miR-193	**↓**		Ameliorates bone resorption
miR-29a	**↓**		Represses osteoclast formation
miR-199a	**↓**		Promotes osteogenic differentiation

*EVs* extracellular vesicles, *OP* osteoporosis

#### Bone to adipose tissue

The OCN-insulin-fat axis exerts an influence on adipose tissue, whereby under normal conditions, adipose tissue displays high insulin sensitivity but becomes a source of insulin resistance [217] (Fig. 3b). However, obesity is associated with several significant health issues, including hypertension, diabetes mellitus, and dyslipidemia, which are the ominous triad linked to insulin resistance. Obesity [20] leads to increased energy metabolism in adipocytes, thereby contributing to these health issues.

The interplay between adipocytes and bone involves complex endocrine, paracrine, and autocrine mechanisms. Adipokines such as leptin and adiponectin play a role in modulating bone remodeling through CNS pathways, adding another layer of complexity. Additionally, the emerging frontier of research focuses on the impact of adipose tissue-derived EVs, particularly those enriched in miR-181a and miR-181b, on osteogenic differentiation and tumorigenesis in MM, offering potential novel therapeutic avenues. Understanding this axis holds clinical significance due to the increasing prevalence of obesity and metabolic syndrome, which are intrinsically linked with bone health. Targeting key regulators like PPARG could provide dual benefits by promoting osteogenesis while inhibiting adipogenesis, thus addressing both aspects of the axis effectively. Moreover, exploring the role of adipose tissue-derived EVs in bone remodeling and disease progression opens up new possibilities for targeted therapies specifically for conditions such as osteoporosis and MM.

### Ovary-bone axis

The primary role of the ovaries in bone metabolism is to secrete estrogen, which directly impacts osteoblasts, osteoclasts, and osteocytes. This disrupts the equilibrium of bone remodeling by promoting bone formation and reducing bone resorption [218] (Fig. 3c). Deficiency of estrogen receptor 1 (ESR1/ERα) specifically in osteoblasts and significantly decreased estrogen levels leads to a reduction in bone trabeculae. Firstly, estrogen impedes RANKL/colony-stimulating factor 1-induced AP-1-dependent transcription by reducing c-Jun expression and phosphorylation. Estrogen also inhibits RANKL-induced osteoclast differentiation [218]. Moreover, estrogen inhibits RANKL-stimulated osteoclastic differentiation by facilitating the complex formation between ERα and breast cancer anti-estrogen resistance 1 (BCAR1). The ERα/BCAR1 complex then sequesters TNF receptor-associated factor 6, leading to reduced NF-κB activation and impaired RANKL-induced osteoclastogenesis [219]. Estrogen has been shown to prolong the lifespan of osteoblasts and inhibit osteoblast apoptosis [220]. At the molecular level, this effect is attributed to estrogen’s activation of the proto-oncogene non-receptor tyrosine kinase (SRC)/adaptor protein 1 (SHC1/SHC)/extracellular signal-regulated kinase (ERK) signaling pathway [220]. Additionally, it involves the downregulation of JNK, which in turn affects the activity of various transcription factors, including cyclic adenosine monophosphate (cAMP)-response element binding protein, CCAAT enhancer binding protein-beta, ETS transcription factor ELK1, and c-Jun/Fos proto-oncogene AP-1 transcription factor subunit [221].

### Testis-bone axis

#### Testis to bone

Testosterone, secreted by the testes, is the primary hormone in males and plays a crucial role in bone metabolism. Androgens bind to androgen receptors on osteoblasts, osteoclasts, osteocytes, and growth plate chondrocytes, to inhibit trabecular bone resorption and maintain bone homeostasis [222, 223]. The impact of testicular function on bone metabolism also relies on the production of two key factors by Leydig cells: insulin-like 3 (INSL3) and cytochrome P450 family 2 subfamily R member 1 (CYP2R1) (Fig. 3d). Specifically, Ferlin et al. [224] elucidated the precise molecular mechanism through which INSL3 exerts its anabolic effects on osteoblasts. When INSL3 activates the G‑protein coupled relaxin family peptide receptor 2 (RXFP2), it initiates a cascade of events including elevation of cAMP levels, activation of the mitogen-activated protein kinase (MAPK) cascade, and upregulation of essential osteoblast genes crucial for osteoblast differentiation, matrix deposition, and osteoclastogenesis, leading to mineralization induction [225]. Additionally, CYP2R1 inactivation in Leydig cells suppresses the expression of 25-hydroxylase [225].

#### Bone to testis

The testicular Leydig cells are primarily influenced by the binding of OCN secreted by osteoblasts to the G protein-coupled receptor class C group 6 member A (Fig. 3d), leading to a positive regulatory effect on testosterone secretion. Specifically, bone exerts its influence through OCN, an osteoblast-derived hormone that binds to specific receptors on Leydig cells and promotes testosterone biosynthesis [226].

The ovary-bone and testis-bone axes represent pivotal hormonal pathways that significantly impact bone metabolism. Estrogen and testosterone, which are the primary hormones secreted by the ovaries and testes, respectively, act as key regulators of osteoblast and osteoclast activity. These axes involve intricate molecular mechanisms such as estrogen signaling mediated by the ERα/BCAR1 complex and testicular function regulated by RXFP2 activation through INSL3. Hormone replacement therapies, already established for postmenopausal osteoporosis treatment, could be further improved by targeting specific molecular pathways such as SRC/SHC/ERK for estrogen or cAMP and MAPK cascades for INSL3. Moreover, the role of vitamin D metabolism, influenced by CYP2R1 in Leydig cells, opens up additional avenues for intervention. However, it is crucial to note that while these hormonal axes offer promising therapeutic targets, they also carry potential risks such as hormone-dependent cancers. Therefore, a nuanced approach considering both the benefits and risks is essential when translating these molecular insights into clinical practice.

### Intestine-bone axis

The gut microbiota (GM), consisting of trillions of microorganisms residing in the intestine and establishing mutually beneficial relationships with the host, primarily influences bone through the intestinal tract [227, 228]. The GM exerts its impact on various physiological processes of the host, including growth, energy metabolism, immune function, and inflammatory processes. Moreover, through nutrient production and absorption, immune regulation, metabolite release, systemic inflammation modulation as well as metabolite generation (Fig. 3e), the GM significantly contributes to the regulation of bone remodeling. Recent studies have shown that the GM potentially regulates bone metabolism by modulating the host’s immune status. Specifically, there is an association between bone metabolism and the balance between Th17 cells [CD4 molecule (CD4)^+^, IL-17A^+^] and T regulatory (Treg) cells [CD4^+^, CD25^+^, forkhead protein P3 (FOXP3)^+^] [229, 230]. Th17 cells are a subset of CD4^+^ T cells that become activated and migrate to the bone stroma where they produce IL-17 to enhance regional inflammation. This elevation in inflammatory cytokines such as TNF-α and IL-6 promotes RANKL expression while activating osteoblast precursor cells to facilitate osteoblast differentiation [229, 231]. On the other hand, Treg cells remain stable in the intestinal mucosa with their development being promoted by several bacteria including *Clostridium* [232], *Bacteroides* [233], *Bifidobacterium* [234], *Lactobacillus* [233], and *Helicobacter* [235]. Among them, the CD4^+^CD25^+^FOXP3^+^ Treg cells suppress T-cell activation through a cytotoxic T lymphocyte-related protein 4-mediated pathway, which reduces the expression of RANKL along with other cytokines thereby inhibiting osteoclast differentiation leading to attenuation of bone resorption while promoting bone formation.

Moreover, increasing evidence emphasizes the significance of the interplay between the GM and bone through innate immune signaling pathways involving receptors such as nucleotide-binding oligomerization domain containing 1/2 (NOD1/2) and TLR5. Germ-free mice lacking NOD1 or NOD2 do not exhibit the typical increase in bone mass or decrease in TNF-α and RANKL expression observed in mice with intestine colonization, suggesting that NOD1 and NOD2 signaling are crucial for the interaction between microbes and bone [236]. TLR5 is known to activate RANKL expression and promote osteoclast formation [237].

Furthermore, the GM produces metabolites that affect bone metabolism, such as short-chain fatty acids (SCFAs; e.g., acetate, butyrate, and propionate), trimethylamine-N-oxide (TNO), and tryptophan (Try). SCFAs not only foster regulatory T-cell development [238, 239] but also suppress Th17 cell generation in the small intestine [240, 241]. They also decrease the production of inflammatory cytokines, including IL-23, IL-17, and IL-6 [240, 241], contributing to systemic immune homeostasis. Moreover, SCFAs inhibit osteoclast formation by inhibiting HDACs essential for human osteoclastic bone formation [242–244]. SCFAs are vital in bone formation and mineralization by modulating the OPG and Wnt signaling pathways [245, 246].

TNO, a GM-dependent metabolite derived from trimethylamine, not only promotes adipogenesis in BMSCs and reduces osteogenesis but also upregulates the expression of pro-inflammatory cytokines (e.g., IL-1β, IL-6, and TNF-α) and reactive oxygen species [247]. Moreover, Try metabolites, particularly kynurenine (KYN) and serotonin, are closely associated with bone metabolism. KYN has been specifically demonstrated to impact BMSC proliferation by directing them toward the osteoblastic cell lineage [248, 249]. Additionally, both osteoblasts and osteocytes possess serotonin receptors [250] that stimulate osteoblast proliferation while inhibiting osteoclast formation upon serotonin binding to these receptors [251].

Probiotics are living microorganisms that exert positive effects on the host’s health when consumed in specific quantities, and they are anticipated to be utilized for the modification of bone disease [252]. *Bacteroides vulgatus* ATCC 8482 inhibited intestinal dysbiosis and downregulated the colonic interferon regulatory factor 6/TLR4/NF-κB pathway, thereby reducing serum TNF-α levels [253]. Similarly, administration of a combination of *Lactobacillus plantarum* NK3 and *Bifidobacterium longum* NK49 suppressed the NF-κB/TNF-α pathway [254]. All three bacteria have shown potential in promoting bone formation. Probiotics have also demonstrated efficacy in preventing bone loss in animal models such as rats and zebrafish [255, 256]. In vitro experiments have further revealed that exposure to *Lactobacillus plantarum* GKM3 and *Lactobacillus paracasei* GKS6 significantly upregulates the expression of osteoblast-related marker genes while downregulating the expression of osteoclast-related genes [257]. Clinical trials have indicated that long-term probiotic supplementation in postmenopausal women reduces serum markers related to bone resorption, thus contributing to alleviating bone resorption [258, 259]. *Akkermansia muciniphila* promotes fracture healing by decreasing intestinal permeability and attenuating inflammatory responses, inducing the release of platelet-derived growth factor-BB, and facilitating H-vessel formation in bone scar tissue [260].

### Pancreas-bone axis

#### Pancreas to bone

Osteoblasts are rich in insulin receptors (INSR) that bind with insulin to regulate bone metabolism (Fig. 3f). Specifically, β-cells in the pancreas secrete insulin, which can interact with the INSR in osteoblasts. Insulin signaling in osteoblasts has the ability to suppress gene expression and promote OCN expression [261]. EVs released by pancreatic cancer cells can transfer microRNAs and other RNAs to bone marrow cells, leading to the downregulation of transcription factor genes associated with the differentiation, polarization, cytokine production, and migration of monocytes and macrophages in the bone marrow. Consequently, this transfer process influences the functionality and metabolic activity of bone marrow cells, ultimately contributing to the establishment of a tumor-promoting microenvironment [262].

#### Bone to pancreas

There are two forms of OCN, fully carboxylated and incompletely carboxylated, which exert their regulatory effects on the pancreas to modulate β-cell and insulin secretion (Fig. 3f). Specifically, OCN functions as a hormone that promotes the proliferation of β-cells and enhances both insulin expression and secretion. OCN also augments insulin sensitivity and promotes energy expenditure [20, 263].

### Thyroid-bone axis

The thyroid primarily affects bone through the secretion of thyroid hormone (TH), which exists in two forms: 3,5,30-L-triiodothyronine (T3) and 3,5,30,50-tetraiodothyronine (thyroxine, T4). T4 is predominantly secreted by the thyroid gland, whereas T3 is mainly derived from the conversion of T4 by type 1 deiodinase in the liver, although a small amount is also secreted by the thyroid gland itself. TH plays a precise regulatory role in bone metabolism, and its deficiency or excessive activity can lead to bone defects (Fig. 3g). Specifically, TH exerts its effects on bone primarily through nuclear TH receptor beta (THRB/TRβ), which exhibits an expression level in skeletal tissues more than 10-time higher than that of nuclear TH receptor alpha (THRA/TRα) [264, 265].

Numerous studies conducted on mice have revealed that those with TRα deficiency or mutation maintain euthyroid status systemically but display phenotypic characteristics resembling juvenile hypothyroidism such as reduced bone formation and decreased BMD [265, 266]. Young mice with TRβ deletions or mutations exhibit typical hyperthyroidism-induced effects on bone resulting in a short stature phenotype [266]. Similarly, adult mice with TRα deficiency or mutation remain euthyroid systemically but demonstrate a significant increase in trabecular volume and mineralization due to a net decrease in osteoclast absorption of bones [265]. In contrast, adult mice with TRβ mutation experience increased osteoclast absorption of bones and severe osteoporosis due to the effects of hyperthyroidism on skeletal cells expressing TRα [265].

In clinical practice, hypothyroidism in mature individuals leads to decreased bone turnover and impaired osteoclast-mediated bone resorption as well as osteoblast-driven bone formation [267]. Simultaneously, the prolongation of the bone remodeling cycle contributes to prolonged secondary mineralization, thereby increasing the susceptibility to fractures in patients [268, 269]. Furthermore, individuals with thyrotoxicosis and hyperthyroidism exhibit an increased risk of fragility fractures, reduced BMD, and osteoporosis [267, 269].

The intricate interplay between various organ systems and bone metabolism is highlighted by the axes of intestine-bone, pancreas-bone, and thyroid-bone. For example, the GM not only influences nutrient absorption but also modulates immune responses that can either promote or inhibit bone resorption. Similarly, insulin from the pancreas and TH, directly and indirectly, impacts bone homeostasis by affecting both osteoblast and osteoclast activity. The potential of intestine-bone axis in modulating bone health through dietary interventions or probiotic supplementation is particularly intriguing, targeting specific microbiota such as *Clostridium* or *Bacteroides* that promote Treg cell development. The pancreas-bone axis offers a compelling avenue for understanding the metabolic underpinnings of bone health, especially in relation to diabetes and pancreatic cancer. With its well-defined hormonal pathways, the thyroid-bone axis provides a robust framework for therapeutic interventions, particularly for conditions like hyperthyroidism that lead to bone loss. However, it is crucial to consider the multifactorial nature of bone metabolism as single-axis interventions may not yield the desired outcomes due to compensatory mechanisms in other axes. Therefore, adopting a holistic multitargeted approach may prove more effective.

## Summary of bone-organ axes

**Fig. 4:**
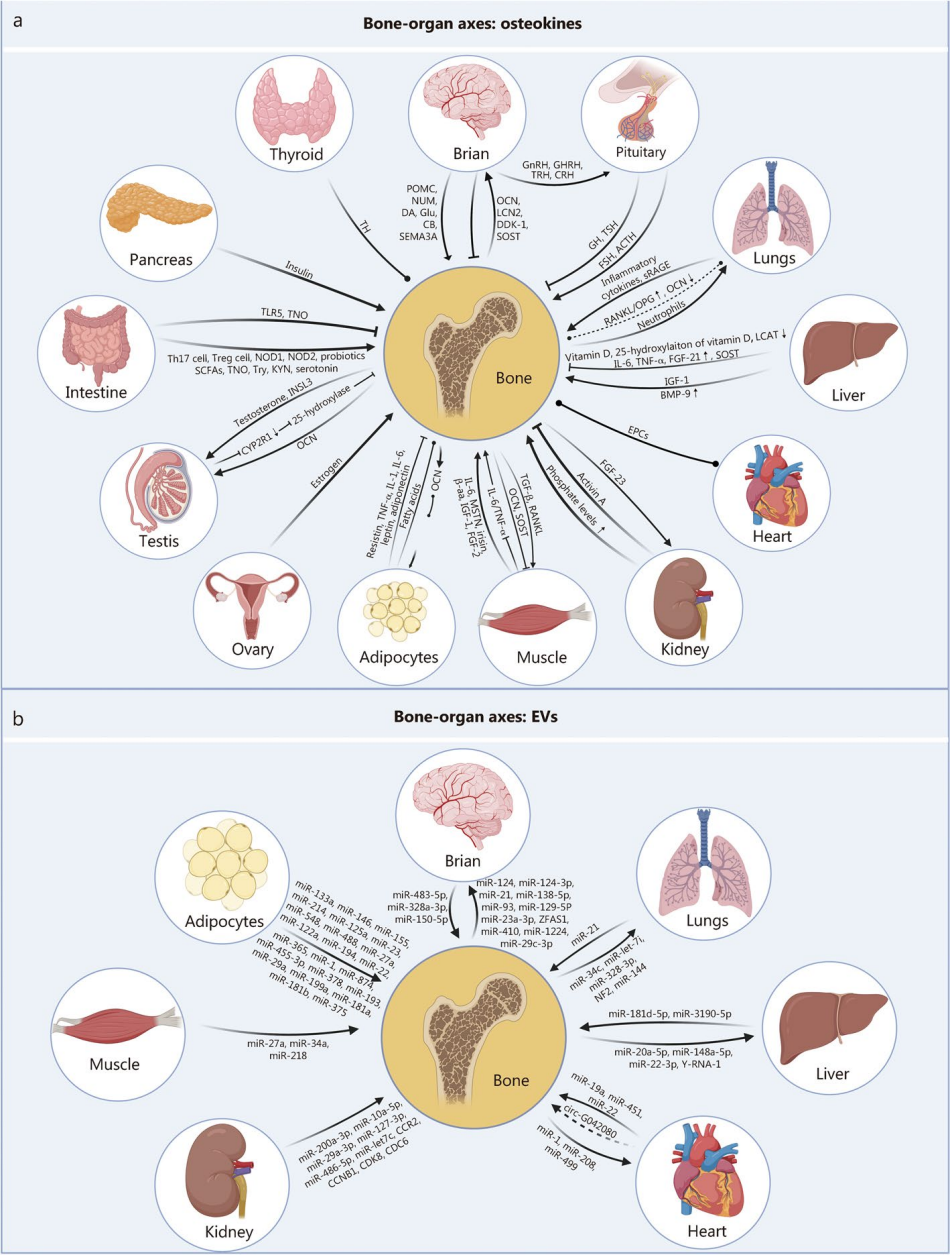
The bidirectional bone-organ crosstalk mediated by osteokines and extracellular vesicles (EVs). **a** The central role of bones in the complex process of interorgan dialogue illustrates the osteokine network that intricately links bone physiology to distal organ systems. The hormone testosterone, for instance, flows from the testis to the bone, influencing its density, while bone-derived OCN reciprocates by guiding muscle metabolism. The illustration also highlights how the kidneys modulate bone health through mineral balance and how the bone, in return, affects renal function through FGF-23. The brain, heart, and adipose tissue are similarly intertwined with bone through their respective hormonal and cytokine emissaries, creating a dynamic equilibrium essential for maintaining overall body health. **b** The figure illustrates the role of EVs in traversing the body, carrying cargoes with potential therapeutic implications for heart, liver, lung, and other diseases. The figure also depicts how EVs from the brain, muscle, kidney, lung, and fat cells directly influence the bone formation process under both physiological and pathological conditions. Collectively, these visual representations elucidate the complex communication networks in which osteokines and EVs are crucial, orchestrating bidirectional bone-organ crosstalk that maintains physiological homeostasis and responds to pathological challenges. GnRH gonadotropin-releasing hormone, GHRH growth hormone-releasing hormone, TRH thyrotropin-releasing hormone, CRH corticotropin-releasing factor, NPY neuropeptide Y, CART cocaine amphetamine-regulated transcript, 5-HT 5-hydroxytryptamine, SEMA4D semaphoring 4D, SEMA3A semaphoring 3A, POMC proopiomelanocortin, NMU neuromedin U, DA dopamine, Glu glutamate, CB cannabinoid, OCN osteocalcin, LCN2 lipocalin 2, DKK1 Dickkopf 1, SOST sclerostin, GH growth hormone, TSH thyroid stimulating hormone, FSH follicle-stimulating hormone, ACTH adrenocorticotropic hormone, IL-1/6/8 interleukin-1/6/8, TNF-α tumor necrosis factor-α, BMP-9 bone morphogenetic protein-9, IGF-1 insulin-like growth factor-1, LCAT lecithin-cholesterol acyltransferase, RANKL receptor activator of nuclear factor kappa-B ligand, OPG osteoprotegerin, sRAGE soluble receptors for advanced glycation end products, PTH parathyroid hormone, FGF-21/23 fibroblast growth factor-21/23, EVs extracellular vesicles, MSTN myostatin, β-aa β-aminoisobutyric acid, TGF-β transforming growth factor-β, RANKL receptor activator of nuclear factor kappa-B ligand, INSL3 insulin-like 3, CYP2R1 cytochrome P450 family 2 subfamily R member 1, Th17 T helper 17, NOD1/2 nucleotide-binding oligomerization domain 1/2, SCFAs short-chain fatty acids, TNO trimethylamine-N-oxide, Try tryptophan, KYN kynurenine, TLR5 Toll-like receptor 5, TH thyroid hormone, miR microRNA

The understanding of bone-organ crosstalk has significantly advanced in recent years. This process can be traced back to the discovery of osteokines, which are cytokines secreted by bone and act as communication agents with distant organs. This concept originated from the identification of specific osteokines and their effects on various organs, such as the kidneys, pancreas, and cardiovascular system (Fig. 4a). The list of osteokines continues to expand, with notable ones including FGF-23, SOST, and OCN. Concurrently, the role of bone as a responsive organ has been highlighted, with various tissues influencing bone homeostasis through their secretion of cytokines. For example, MSTN from muscles and leptin from adipose tissues have significant impacts on bone physiology [270]. Notably, several osteokines exhibit autocrine effects on the bone itself. The exploration of bone-derived factors and their roles in health and disease is ongoing. Currently, there is a primary focus on investigating the roles of osteokines in mediating communication between bone and other organs as well as their implications in skeletal disorders, metabolic diseases, and aging processes. Discovering novel osteokines and understanding their specific functions could potentially pave new paths for therapeutic interventions targeting these diseases. Furthermore, the identification of these osteokines could serve as valuable biomarkers for monitoring various physiological responses and disease progression.

In addition, the perspective on cellular communication and the role of EVs have significantly evolved in recent years. EVs carry a complex cargo consisting of proteins, lipids, and nucleic acids, which reflect their originating cell and convey information to recipient cells [271]. This concept was further reinforced by the discovery of EVs involvement in various physiological and pathological processes, such as angiogenesis, cancer metastasis, and immune responses. Emerging research has identified numerous EV-associated molecules, including specific microRNAs, proteins, and lipids [272]. Moreover, the role of EVs extends to various organs through autocrine, paracrine, or endocrine mechanisms facilitating intricate interactions between cells within both local and distant organs [6, 273]. Bone-derived EVs are known to interact with the immune system, kidneys, and even the CNS (Fig. 4b). Recently discovered exercise-induced EVs suggest their potential role in mediating some systemic benefits derived from physical activity. Despite the predominant focus on studying EVs in cancer biology and immune responses, exploring their significance in exercise physiology and organ crosstalk is an emerging field of interest [274]. It is imperative to investigate methods for determining specific vesicle origins while elucidating their precise actions and mechanisms when targeting different organs in vivo. Identifying novel EV-associated molecule specificities and their specific roles could lead to groundbreaking therapeutic targets as well as diagnostic tools for various diseases, thereby revolutionizing our comprehension of intercellular communication dynamics alongside systemic homeostasis.

## Implications for bone-on-chips and assembloids

As previously mentioned, significant achievements have been made in understanding the bidirectional communication between bone and other organs through the utilization of animal models and clinical studies over the past few decades. However, there still exist numerous interorgan connections that pose challenges for further exploration due to discrepancies between model animals and humans as well as inadequate clinical models. Multiple diverse biotechnologies have emerged to address these issues and provide novel solutions for biology and clinical research.

The organ-on-a-chip technology replicates physiological microenvironments to mimic the intricate interactions between different tissues within the body [275]. It provides a dynamic platform for unraveling the complex network of biochemical signals and mechanical forces that govern bone-organ axes. By allowing manipulation and monitoring of these interactions within a controlled, physiologically relevant context, organ-on-a-chip technology elucidates the underlying mechanisms of bone-organ crosstalk in health and disease. Furthermore, advancements in organ-on-a-chip technology inherently rely on a profound understanding of bone-organ axes. The more we comprehend these axes, the more precise and effective our development of organ-on-a-chip models becomes.

**Fig. 5:**
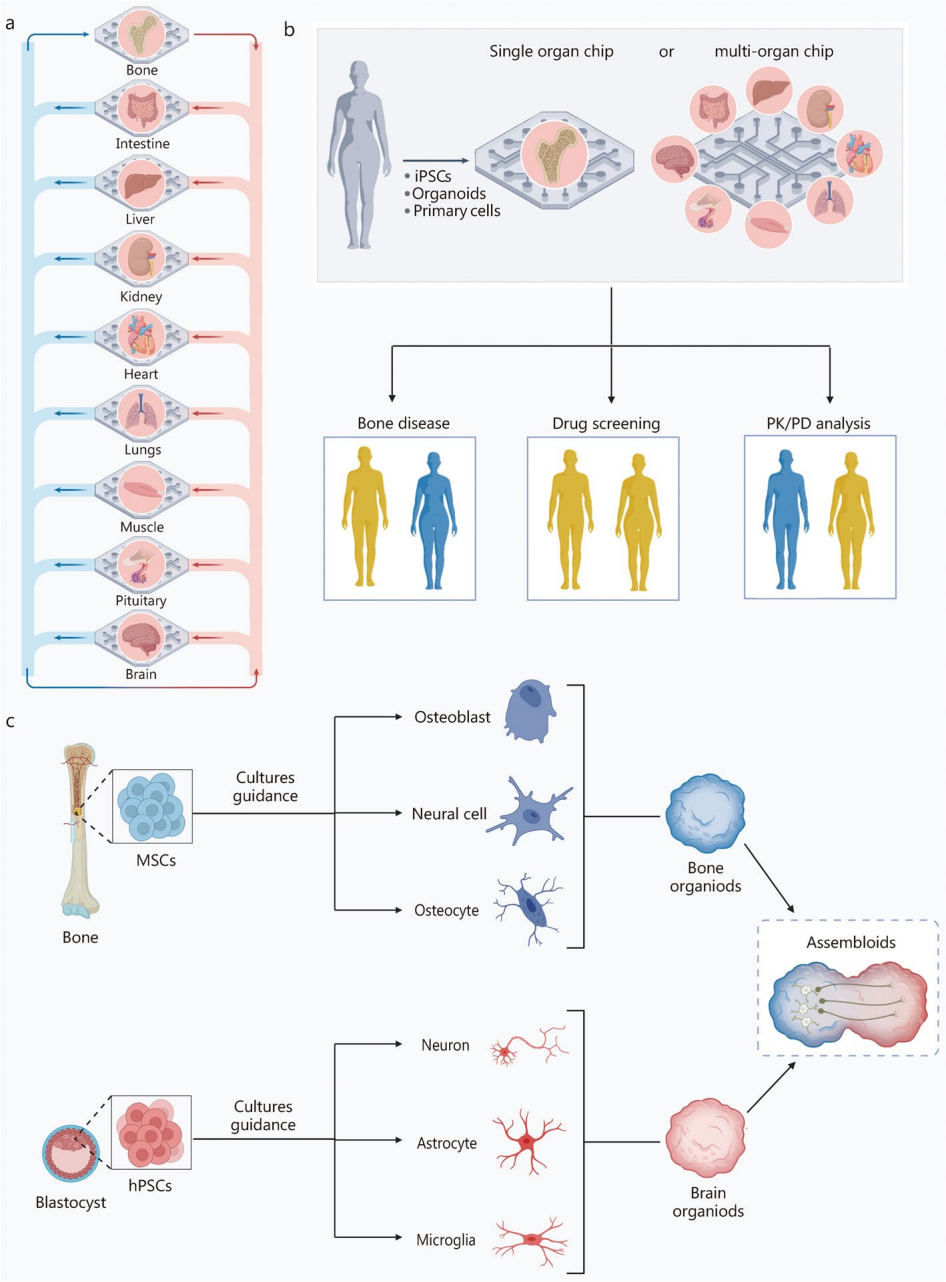
The implications of bone-on-chips and assembloids in bone-organ axes. **a** Organ-on-a-chip system can simulate the physiological connections of organs in our bodies by simulating blood circulation through ordinary flow channels. **b** The application of bone-on-chips in unraveling the mechanism of bone disease, drug screening, and PK/PD analysis. **c** Assembloids integrated with bone and brain organoids. iPSCs induced pluripotent stem cell, PK/PD pharmacokinetic/pharmacodynamic, hPSCs human pluripotent stem cells

The integration of two or more organs-on-a-chip in a dynamic fluid system enables the simulation of complex physiological and pathophysiological responses, as well as drug compound delivery, absorption, distribution, metabolism, and excretion (Fig. 5a). Bone-on-chip is a microarray-derived from induced pluripotent stem cells (iPSCs), primary cells, or organoids [276, 277] that can replicate the bone’s physiological microenvironment and study bone-related diseases such as bone cancer, bone tumors, osteoporosis. It is used for research on bone disease models, drug screening, and pharmacokinetic/pharmacodynamic (PK/PD) analysis (Fig. 5b). For example, to simulate bone disease models, Chou et al. [278] designed a primary human vascularized bone marrow chip with top and bottom channels separated by a porous membrane. In the top channel, CD34^+^ cells and stromal cells derived from the bone marrow were cocultured within a 3D ECM gel. The bottom channel consisted of parallel channels lined with human vascular endothelial cells perfused with the culture medium. Vascularized bone marrow microarrays inoculated with CD34^+^ cells, BMSCs, and vascular endothelial cells could sustain various blood cell lineages for over one month in culture. They can replicate aspects of bone marrow injury, including granulocyte toxicity after exposure to clinically relevant chemotherapeutic agents or ionizing radiation and bone marrow recovery after drug-induced myelosuppression. They can also serve as an in vitro model of hematopoietic dysfunction.

In the field of drug screening research, McAleer et al. [279] developed a pump-free, reconfigurable, multiorgan-on-a-chip system that utilizes recirculating serum-free media to predict the preclinical efficacy of drug targeting, metabolic conversion, and off-target toxicity. This innovative system incorporates functional bio-microelectromechanical systems. Primary human hepatocytes were co-cultured with two cancer-derived human bone marrow cell lines (Kasumi-1 myeloblasts and MEG-01 megakaryocytes) to examine the effects of antileukemia drugs. Their study revealed that diclofenac and imatinib exhibited cytostatic effects on myeloma proliferation. Imatinib did not show any adverse effects on liver viability; however, diclofenac reduced liver survival by 30%. For PK/PD analysis, Herland et al. [280] employed vascular endothelium-lined channel fluid-coupled bone marrow, liver, and kidney chips to establish a human model for drug absorption, metabolism, and excretion model of cisplatin (a cancer chemotherapy agent), enabling prediction of human PK parameters and PD responses. This multiorgan chip included an arteriovenous reservoir where cisplatin was administered to stimulate intravenous injection.

Assembloids are 3D aggregates of multiple organoids [281]. Their creation involves the controlled combination of different cell types, such as cell lines, primary cells, or iPSCs, to promote self-organization and the formation of tissue-like structures [282]. Assembloids provide researchers with a controlled environment to explore complex cellular behaviors, cell signaling pathways, and tissue development. Through in vitro integration of bone and other organoids (e.g., brain organoids), assembloids offer an unprecedented opportunity to delve into the cellular crosstalk and tissue-tissue interactions underlying bone-organ axes (Fig. 5c). Moreover, advancements in assembloid technology have been influenced by the exploration of bone-organ axes. Insights into the cellular and molecular mechanisms of bone-organ axes enable the development of more sophisticated and accurate assembloid models.

Both technologies are powerful tools for predicting and evaluating pathophysiological changes in bone-organ axes, providing a robust platform for developing and optimizing therapeutic strategies for bone-related and systemic diseases. By advancing our understanding of the etiology and progression of bone-organ axis diseases, these technologies lay the foundation for novel diagnostic and therapeutic approaches. In essence, the synergistic integration of organ-on-a-chip and assembloid technologies with the investigation of bone-organ axes holds immense potential to revolutionize our comprehension of bone-organ interactions. This interplay between research and technology establishes a robust groundwork for devising innovative strategies aimed at preventing, diagnosing, and treating both bone-associated and systemic diseases.

## Conclusions

The bone-organ axes, characterized by bidirectional communication via osteokines, EVs, hormones, and metabolites, are crucial in maintaining homeostasis and functionality across diverse organ systems. This review emphasizes the intricate crosstalk between bone and various organs, highlighting its potential implications for disease pathogenesis and treatment. However, systematically exploring these complex interrelationships remains an essential frontier in biomedical research. Many factors, mechanisms, and EVs involved in organ interactions still need to be investigated due to the limitations of existing experimental technologies and the inadequacy of the research methodologies. The underlying mechanisms governing the interactions between Alzheimer’s disease and bone as well as the effects of lung cancer and bone injury are yet to be elucidated. Additionally, further investigation is needed to understand the mechanisms behind changes in bone mass observed in chronic liver disease.

To address these gaps in knowledge, we propose utilizing advanced techniques such as bone-on-chips, organoids, and assembloids that can simulate human organ-to-organ communication in vitro. These innovative approaches hold great promise for breakthroughs in scientific research. Furthermore, employing these new biotechniques can also facilitate investigations into factors or EVs that have been studied at cellular or animal levels with the aim of achieving significant advancements in disease treatment. However, it should be noted that there is ongoing controversy within the international research community regarding the pathology/physiology of EVs studied under laboratory conditions. Future investigations using advanced techniques will undoubtedly provide more insights into the intricate dynamics of bone-organ axes while paving the way for novel therapeutic strategies and an enhanced understanding of organismal physiology.

## Data Availability

Not applicable.

## Abbreviations

ACTH: Adrenocorticotropic hormone
AKI: Acute kidney injury
Akt: Protein kinase B
ALI: Acute lung injury
AP-1: Activator protein 1
BBB: Blood–brain barrier
BCAR1: Breast cancer anti-estrogen resistance 1
BMD: Bone mineral density
BMP-9: Bone morphogenetic protein-9
BMSC-EVs: Extracellular vesicles derived from bone marrow mesenchymal stem cells
BMSCs: Bone marrow mesenchymal stem cells
cAMP: Cyclic adenosine monophosphate
CART: Cocaine amphetamine-regulated transcript
CB: Cannabinoid
CCL2: C–C motif chemokine ligand 2
CCR2: C–C motif chemokine receptor 2
CF: Cystic fibrosis
CKD: Chronic kidney disease
CNS: Central nervous system
COPD: Chronic obstructive pulmonary disease
COVID-19: Chronic coronavirus disease 2019
CVDs: Cardiovascular diseases
CYP2R1: Cytochrome P450 family 2 subfamily R member 1
DA: Dopamine
DKK1: Dickkopf 1
ECM: Extracellular matrix
ENaC: Epithelial sodium channel
EPCs: Endothelial progenitor stem cells
EVs: Extracellular vesicles
FGF-21: Fibroblast growth factor-21
FSH: Follicle-stimulating hormone
GH: Growth hormone
Glu: Glutamate
GM: Gut microbiota
HIBD: Hypoxic-ischemic brain damage
HOD: Hepatic osteodystrophy
IL-1: Interleukin-1
INSL3: Insulin-like 3
INSR: Insulin receptors
JNK: Janus kinase
KYN: Kynurenine
LCAT: Lecithin-cholesterol acyltransferase
LCN2: Lipocalin 2
LRP5: Low-density lipoprotein receptor-related protein 5
MAPK: Mitogen-activated protein kinase
MARCKS: Myristoylated alanine-rich protein kinase C substrate
MBD: Mineral and bone disease
MC4R: Melanocortin 4 receptor
MCAO: Middle cerebral artery occlusion
miR: MicroRNA
MM: Multiple myeloma
MSC: Mesenchymal stem cell
MSTN: Myostatin
NF-κB: Nuclear factor kappa-B
NMU: Neuromedin U
NOD1/2: Nucleotide-binding oligomerization domain containing 1/2
NPY: Neuropeptide Y
NSCLC: Non-small cell lung cancer
OCN: Osteocalcin
OLT: Orthotopic liver transplantation
OP: Osteoporosis
OPG: Osteoprotegerin
OSX: Osterix
PGE2: Prostaglandin E2
PI3K: Phosphoinositide 3-kinase
PK/PD: Pharmacokinetic/pharmacodynamic
POMC: Proopiomelanocortin
PPARG: Peroxisome proliferator-activated receptor gamma
PTEN: Phosphatase and tensin homolog
PTH: Parathyroid hormone
RANKL: Receptor activator of nuclear factor kappa-B ligand
RUNX2: Runt-related transcription factor 2
RXFP2: Relaxin family peptide receptor 2
SARS-CoV-2: Severe acute respiratory syndrome coronavirus 2
SCFAs: Short-chain fatty acids
Semas: Semaphorins
SOST: Sclerostin
TBI: Traumatic brain injury
TH: Thyroid hormone
TLR4/5: Toll-like receptor 4/5
TNF-α: Tumor necrosis factor-α
TNO: Trimethylamine-N-oxide
Treg: T regulatory
TRH: Thyrotropin-releasing hormone
Try: Tryptophan
TSH: Thyroid stimulating hormone
β-aa: β-aminoisobutyric acid
5-HT: 5-hydroxytryptamine/serotonin
